# A genome-wide study of Hardy–Weinberg equilibrium with next generation sequence data

**DOI:** 10.1007/s00439-017-1786-7

**Published:** 2017-04-03

**Authors:** Jan Graffelman, Deepti Jain, Bruce Weir

**Affiliations:** 1 Department of Statistics and Operations Research, Universitat Politècnica de Catalunya, Avinguda Diagonal 647, 08028 Barcelona, Spain; 2 Department of Biostatistics, University of Washington, University Tower, 15th Floor, 4333 Brooklyn Avenue, Seattle, WA 98105-9461 USA

## Abstract

Statistical tests for Hardy–Weinberg equilibrium have been an important tool for detecting genotyping errors in the past, and remain important in the quality control of next generation sequence data. In this paper, we analyze complete chromosomes of the 1000 genomes project by using exact test procedures for autosomal and X-chromosomal variants. We find that the rate of disequilibrium largely exceeds what might be expected by chance alone for all chromosomes. Observed disequilibrium is, in about 60% of the cases, due to heterozygote excess. We suggest that most excess disequilibrium can be explained by sequencing problems, and hypothesize mechanisms that can explain exceptional heterozygosities. We report higher rates of disequilibrium for the MHC region on chromosome 6, regions flanking centromeres and p-arms of acrocentric chromosomes. We also detected long-range haplotypes and areas with incidental high disequilibrium. We report disequilibrium to be related to read depth, with variants having extreme read depths being more likely to be out of equilibrium. Disequilibrium rates were found to be 11 times higher in segmental duplications and simple tandem repeat regions. The variants with significant disequilibrium are seen to be concentrated in these areas. For next generation sequence data, Hardy–Weinberg disequilibrium seems to be a major indicator for copy number variation.

**Electronic supplementary material:**

The online version of this article (doi:10.1007/s00439-017-1786-7) contains supplementary material, which is available to authorized users.

## Introduction

The Hardy–Weinberg law is a fundamental population-genetic principle expressing that the genotypes *AA*, *AB* and *BB* of a bi-allelic genetic marker are expected to occur with relative frequencies $p^2$, 2*pq* and $q^2$, *p* and *q* being the *A* and *B* allele frequencies, respectively. The law rests on many assumptions including sexual reproduction with random mating, non-overlapping generations, negligible mutation and migration rates, equality of allele frequencies in the sexes, absence of natural selection and absence of genotyping errors, which are usually discussed at length in genetic textbooks (Crow and Kimura 1970; Hartl 1980). In this paper, we tacitly assume that most assumptions are at least approximately met, and mainly focus on genotyping problems as a potential source for deviation from equilibrium.

In modern large-scale genotyping studies, genetic markers are typically tested for Hardy–Weinberg proportions (HWP) by using exact test procedures (Wigginton et al. 2005). Next generation sequencing (NGS) data are especially prone to genotyping error when relying on low-coverage sequencing (Nielsen et al. 2011). Genotyping error is a common cause for disequilibrium, and by testing markers for HWP, problematic markers can be identified (Gomes et al. 1999; Hosking et al. 2004; Leal 2005; Teo et al. 2007). In many genotyping studies, markers are filtered on the basis of their *p* value in a HW test prior to subsequent analysis, with the idea to create high-quality data-sets from which (hopefully) most genotyping error has been removed. We think it is important to understand and identify the causes of disequilibrium, rather than merely discarding the significant results. Hence, we evaluated variants by also taking into account their genomic context, position, pertinence to a region with repetitive DNA, read depth and other factors. Plotting HW test results against the chromosomal position helped us identify genomic regions where variants with significant disequilibrium were clustered and thus enabled us to systematically investigate the cause(s) of disequilibrium. The structure of this article is as follows. First, we give a brief description of the database used and summarize exact tests for HWP for the autosomes and the sex chromosomes. Second, the Results section reports on global results, and contains subsections that focus on particular areas with an exceptional rate of HWD. Finally, discussion and conclusions complete the paper.

## Database and methods

### Database

We used data from the Japanese sample [in Tokyo, Japan (JPT)] of phase 3 of the 1000 Genomes project (2015). We used the JPT sample for being relatively homogeneous without closely related individuals. The sample consisted of 104 unrelated individuals (56 males and 48 females). Variant call data from whole genome sequencing of these individuals mapped to reference genome GRCh37 (hg19) were downloaded from http://www.internationalgenome.org/. We excluded all variants that had no RS identifier, duplicated identifiers, variants with an identical base pair position and variants with more than 5% missing values. This filtering retained 82,379,719 variants from all 22 autosomes and the X-chromosome (see Table 1). Monomorphic variants, which constituted 85% of all variants, were further filtered leaving 12,455,090 variants for HW computations. All autosomal variants were tested for HWP by a standard exact test, and X-chromosomal variants were tested by an omnibus exact test for HWP in females and equality of allele frequencies in males and females (Graffelman and Weir 2016). We took special care to test variants in the pseudo-autosomal regions (PAR1 and PAR2) of the X-chromosome with the autosomal exact test. However, because we restricted the analysis to variants with an RS identifier, there was only one variant in the PAR1 region and no variants in the PAR2 region. We used Plink (Purcell et al. 2007) for basic data manipulation and the R package Hardy–Weinberg (Graffelman 2015) for statistical testing. For the sake of comparison, a second sample of the 1000 Genomes project taken from the Yoruba (YRI) population was submitted to the same analysis, and the corresponding results are included in Supplementary Appendix B.

### Methods

There are several statistical methods available for testing markers for HWP, such as the Chi-square test, the likelihood ratio test, the exact test and the permutation test. The various tests are summarized by Weir (1996). The Chi-square test has long been the most popular test for HWP, but with modern computing power it is feasible to obtain exact test results on a genome-wide scale. The autosomal exact test is based on the distribution of the number of heterozygotes given the observed count of the minor allele, say *A* ($N_{AB}|N_A$). Under the assumption of HWE, for bi-allelic autosomal markers with alleles *A* and *B*, this distribution is given by:

1 $$\begin{aligned} P \left( N_{AB}|N_A \right) = \frac{n_A! n_B! n! 2^{n_{AB}}}{n_{AA}! n_{AB}! n_{BB}! (2n)!}, \end{aligned}$$

where $n_A$ and $n_B$ represent the sample allele counts, $n_{AA}, n_{AB}$ and $n_{BB}$ the sample genotype counts, and *n* the sample size. The standard *p* value of the exact test is the probability of the observed sample plus the sum of the probabilities of all possible samples that are less likely than the observed sample. We use a two-sided test because there is, a priori, no reason to expect an excess or a deficiency of heterozygotes. It is known that, due to the discreteness of the data, the distribution of the *p* values obtained by using this test is not uniform under the null of HWE (Rohlfs and Weir 2008; Wigginton et al. 2005). We therefore used the exact mid *p* value (Graffelman and Moreno 2013), now also available in the Plink program (Purcell et al. 2007), which has expectation 0.5 under the null, and, more importantly, provides for a test that has its rejection rate close to the nominal level.

In HWP tests for markers on the X-chromosome, hemizygous males are usually discarded, but recent methodological advances (Graffelman and Weir 2016) have made it possible to include males in an exact test on HWE for the X-chromosome. The X-chromosomal exact test is an omnibus test that simultaneously tests HWP in females and equality of male and female allele frequencies. The X-chromosomal exact test is based on the joint distribution of the number of A males and the number of heterozygote females given the A allele count, and this distribution is given by:

2 $$\begin{aligned} \small P \left( M_A, F_{AB}\mid n, n_A, n_m \right) = \frac{n_{A}! n_{B}! n_m! n_f!}{m_A! m_B! f_{AA}! f_{AB}! f_{BB}! n_\mathrm{t}! } 2^{f_{AB}}, \end{aligned}$$

where $n_A$ and $n_B$ represent the sample allele counts, $n_m$ and $n_f$ the number of males and females, respectively, and $m_A, m_B, f_{AA}, f_{AB}$ and $f_{BB}$ the male and female genotype counts. The total number of alleles for an X-chromosomal marker is given by $n_\mathrm{t} = 2 n_\mathrm{f} + n_\mathrm{m}$. Exact *p* values were calculated by using the mid *p* value definition, which is half the probability of the observed sample plus the probabilities of all samples more extreme than the observed sample, according to Eq. (1) for autosomal markers and according to Eq. (2) for markers on the X-chromosome.

The power and Type I error rate of Chi-square and exact tests have been studied in detail by several scholars (Emigh 1980; Wigginton et al. 2005; Graffelman and Moreno 2013) and it is well-known that standard exact tests strictly control the type-1-error rate, but that they are conservative and have low power at low minor allele frequencies. The use of the mid *p* value ameliorates this to some extent. In a genome-wide analysis huge numbers of variants are tested, and inevitably false positives (markers in equilibrium for which the test rejects it) and false negatives (markers out of equilibrium that go unnoticed because the test does not reject) will arise.

## Results

**Fig. 1:**
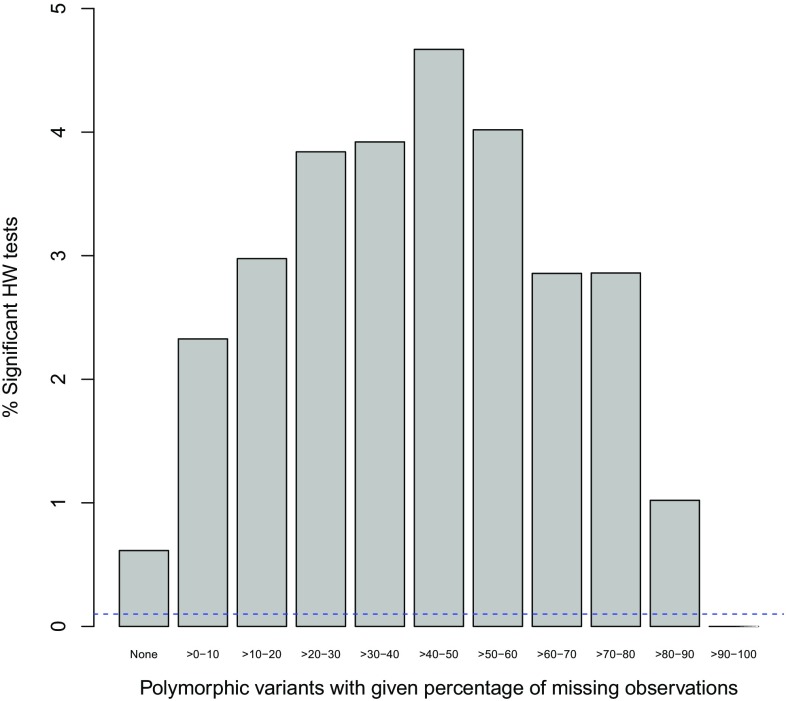
Percentage of significant HW tests for polymorphic autosomal variants as a function of the percentage of missing values at $\alpha = 0.001$. The *horizontal dashed line* corresponds to the HapMap exclusion threshold

In this section, we first report some overall results. Some areas with a high disequilibrium rate are discussed in separate subsections below. We first address the issue of missing values. Figure 1 shows the percentage of significant variants at level $\alpha = 0.001$ in different bins of variants grouped by their percentage of missing values. The figure clearly shows an upward trend, showing that markers with more missing values tend to be more often out of equilibrium. Missing values are indicative of the existence of genotyping error and Fig. 1 therefore affirms the importance of HW testing as a tool to detect genotyping error.

Note that the percentage of significant variants starts to drop for variants with more than 50% missing values. This should not be taken as evidence that markers with that many missing values are better, but is a consequence of a loss of power due to a decreasing sample size. Indeed, a salient feature of Fig. 1 is that in the 0–50% range the number of significant results rises *despite* the ever decreasing power.

Figure 1 shows large proportions of variants with exact *p* values below the significance level and thus clearly shows that there is far more disequilibrium than would be expected by chance alone. The significant markers are typically polymorphic markers that do not have a low minor allele frequency (See supplementary Figure S1). In order to avoid low-quality markers with many missing values and excessive genotyping error, RS variants with more than 5% missings were discarded for the following HW computations. As pointed out in earlier work (Graffelman et al. 2015), with larger numbers of missing values inference on HWP can be biased, and the missing values should be taken into account using statistical techniques like multiple imputation.

**Fig. 2:**
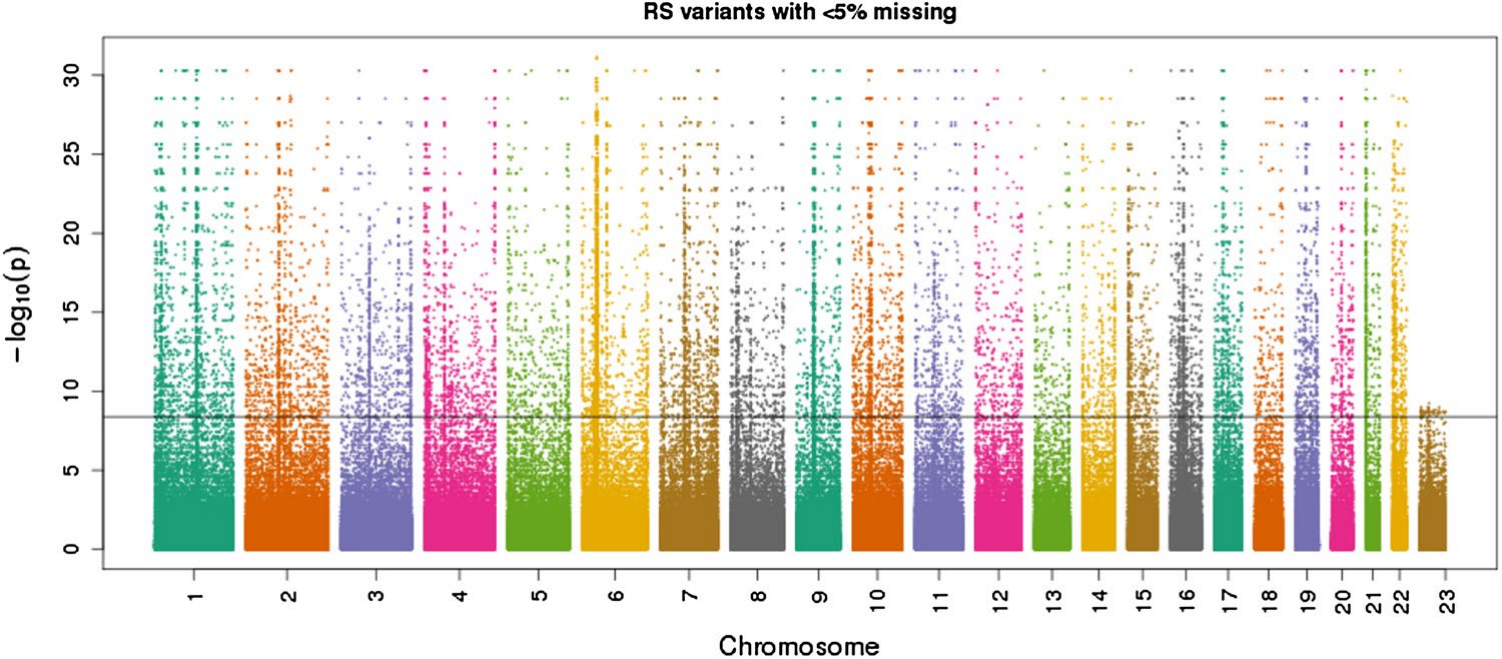
Manhattan plot of exact mid *p* values for Hardy–Weinberg equilibrium of the JPT sample. The *horizontal line* corresponds to the Bonferroni significance threshold ($-log_{10}(0.05/12133408) = 8.4$, using only polymorphic autosomal variants)

We plot exact HW exact *p* values chromosome-wise in a Manhattan plot in Fig. 2. This reveals several regions where variants with significant HWE are clustered. Typically most chromosomes show a long stripe of significant results close to the centromere region. In Fig. 2, the X-chromosome shows fewer transformed *p* values in the range 10–30, whereas for the autosomes many results are observed in that range. For practical purposes, there is little difference between a transformed *p* value of 10 or 30, both being extremely significant. Indeed, if the *p* values had been rounded to 10 decimals, this phenomenon would have gone unnoticed. For the autosomal variants, the tails of Levene–Haldane distribution ($N_{AB}|N_A$) are longer and therefore there is more scope for infinitesimal *p* values in autosomal markers. In other words, due to a reduced number of alleles ($n_\mathrm{t}$ for X-chromosomal, and $2n > n_\mathrm{t}$ for autosomal variants), there is less power to detect disequilibrium on the X-chromosome.

We quantify the amount of disequilibrium in Table 1, where we report number of variants, percentage of monomorphic markers and percentage of polymorphic significant markers at the HapMap exclusion level of $\alpha = 0.001$ per chromosome (The International HapMap Consortium 2007). For each autosome, about 84–86% of all variants with an RS identifier is monomorphic. On the X-chromosome the percentage of monomorphics is somewhat lower. The median of the within-population inbreeding coefficient, calculated over all polymorphic autosomal RS variants, is negative for all chromosomes and varies between ${-0.005}$ and ${-0.01}$, indicating that an excess of heterozygotes is more common than a deficiency of heterozygotes. The autosome-wide median of the inbreeding coefficient is, for the JPT sample, $-0.007$. The rate of significant variants, expressed as the number of significant variants among all polymorphic variants per chromosome, varies from 0.3 to 1.6% which is 3–16 times as much as expected by chance alone. Over all autosomes, 85% is monomorphic, and 0.62% of the polymorphic variants is significant, which is 6.2 times as much as expected by chance alone. Chromosomes 6, 21 and 22 clearly have a higher rate of disequilibrium. Significant disequilibrium is, over all autosomes, in 59% of the cases due to an excess of heterozygotes. Almost all chromosomes show more variants with significant excess than significant deficiency. Chromosome 6, 19 and 22 are the only exceptions. For chromosome 6 heterozygote deficiency is by far more common, and excess accounts for only about 22% of the significant results. For chromosomes 17, 20 and 21 about 80% of the significant results is due to heterozygote excess. Excess disequilibrium also manifests itself in chromosome-wide QQ-plots of the exact test *p* values against the uniform distribution shown in supplementary Figures S2, S3 and S4. These QQ-plots show a horizontal band at mid *p* value 0.5 which is due to low MAF variants, and deviate strongly from uniformity in the lower tail of the *p* value distribution.

**Table 1:** Descriptive statistics for each chromosome and autosome-wide and genome-wide summaries of the JPT sample: number of SNPs (with RS identifier and with less than 5% missing values), percentage monomorphic markers, percentage significant markers in a HW exact test with $\alpha = 0.001$ among the polymorphic markers, percentage of significant markers due to heterozygote excess, median of the inbreeding coefficient (*f*) for all polymorphic variants, median read depth (DP) for all polymorphic variants. Results for the X-chromosome reported for an all-individuals test and for a females-only test

Chr	#SNPs	%Mono	%HWE sig.	%HetExc	Median *f*	Median DP
1	6,448,745	85.00	0.68	75.27	−0.008	18,032
2	7,060,690	85.53	0.48	63.40	−0.005	17,954
3	5,814,755	85.01	0.47	54.07	−0.006	17,944
4	5,715,198	84.77	0.53	56.59	−0.006	17,523
5	5,250,147	85.44	0.35	74.11	−0.010	17,925
6	5,008,031	83.87	1.26	22.29	−0.005	17,811
7	4,702,165	84.91	0.59	57.93	−0.007	17,747
8	4,583,614	85.66	0.46	62.95	−0.007	17,948
9	3,549,967	84.95	0.74	53.12	−0.010	17,919
10	3,979,921	84.69	0.57	68.69	−0.009	18,056
11	4,033,317	85.34	0.49	59.10	−0.008	18,080
12	3,763,369	84.93	0.43	69.52	−0.010	17,960
13	2,849,212	84.72	0.39	64.87	−0.006	17,506
14	2,647,168	84.87	0.57	59.65	−0.005	17,920
15	2,417,253	84.96	0.62	72.02	−0.010	18,210
16	2,689,853	85.66	0.86	73.99	−0.005	18,179
17	2,321,652	85.36	0.71	79.08	−0.005	17,779
18	2,260,418	84.86	0.40	71.13	−0.010	17,841
19	1,825,981	84.22	0.93	48.88	−0.010	17,098
20	1,807,620	85.47	0.44	84.45	−0.010	18,277
21	1,101,960	84.58	1.59	79.61	−0.005	17,733
22	1,100,007	84.29	1.07	44.87	−0.010	17,924
Autosomes	80,931,043	85.01	0.62	58.93	−0.007	17,889
X (all)	1,448,077	77.79	0.32	–	–	13,444
X (fem)	1,448,077	79.97	0.23	81.18	−0.021	13,444
Genome	82,379,719	84.88	0.61			17,802

In the remainder of this section we focus on several areas that show exceptional rates of HWD: the MHC complex, the short arms of acrocentric chromosomes 21 and 22, centromeres, distal ends of p-arms (telomeres), the X-chromosome and some regions with incidental spikes or horizontal stripes. We also address the relationship between HWD and read depth and copy number variation.

### The MHC complex

**Fig. 3:**
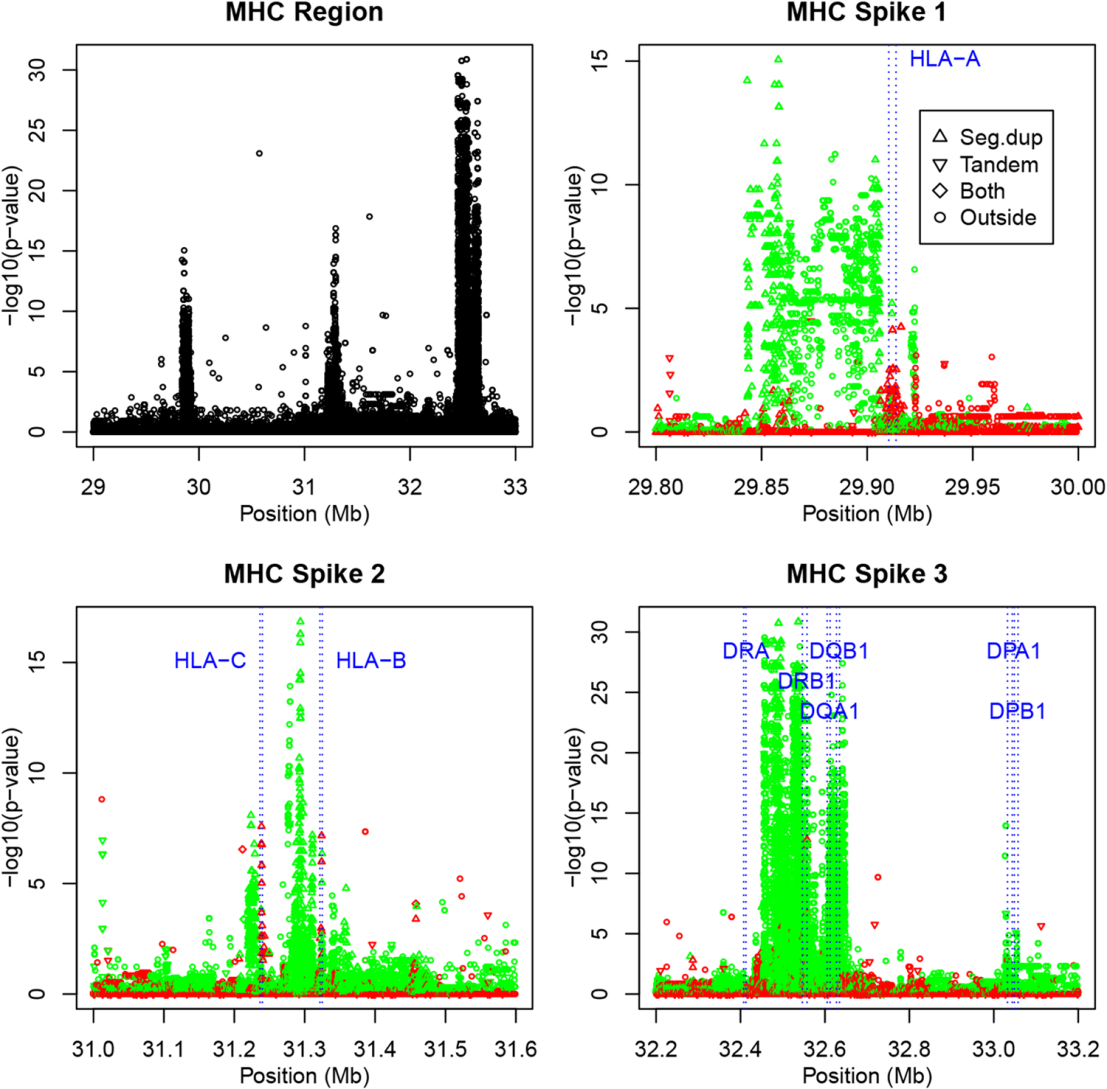
**a** Hardy–Weinberg track showing the exact mid *p* values of tests for disequilibrium for each variant in the MHC region on chromosome 6. **b**–**d** Plots of the exact *p* values for the observed spikes colored according to the sign of the inbreeding coefficient (*green* $f > 0$, *red* $f < 0$), annotated with HLA class I and II genes. *Plotting symbols* indicate if a variant is inside a segmental duplication, inside a tandem repeat, inside both, or outside such regions

The human major histocompatibility complex (MHC) region is well known for its role in the immune system. This extremely gene-dense region covers a region of 3.6 Mb on chromosome 6 (The MHC sequencing consortium 1999). In Fig. 3a we show a Hardy–Weinberg track of a part of chromosome 6 that includes this region. Three very strong (composite) spikes of disequilibrium are observed. This region accounts for the higher overall rate of 1.26% disequilibrium on this chromosome. The MHC is known for the highly polymorphic HLA genes, and is also known to contain duplicated sequences and CNVs (Traherne 2008). If the MHC region is excluded from the HW analysis, the disequilibrium rate on chromosome 6 drops to 0.49% and is comparable to the rates observed on the other autosomes. The descriptive statistics given in Table 1 are repeated in Table 2, but stratified for markers inside and outside the MHC region, and stratified for HLA class I and class II genes. Table 2 shows a much lower rate of monomorphic markers (59.6%) and a much higher rate of HWD (11.9% significant HWD) inside the MHC region. Figure 3b–d shows the observed spikes in higher resolution, with variants with heterozygote deficiency in green, and variants with heterozygote excess in red. This shows that the three spikes mainly correspond to areas of heterozygote deficiency. Significant heterozygote excess is observed only at a few positions. Despite the strong spikes of heterozygote deficiency, the median inbreeding coefficient of the MHC region overall is negative, meaning that there are many non-significant variants with slight heterozygote excess in the region. If the genotyping results in the MHC region are correct, then the conclusion would be that the MHC region contains a considerable set of variants with strong heterozygote deficiency. However, the alternative interpretation is that the region is affected by genotyping error due to duplications (see "Discussion and conclusion").

We annotated the Hardy–Weinberg track of the MHC region in Fig. 3 with the position of the HLA class I genes (HLA-A, HLA-B and HLA-C) and six HLA class II genes (DPA1, DPB1, DQA1, DQB1, DRA and DRB1) obtained by consulting the NCBI database (https://www.ncbi.nlm.nih.gov/). This shows that strong spikes of heterozygote deficiency occur upstream of the HLA-A, downstream the HLA-B and downstream the HLA-C genes. The variants *inside* these genes themselves appear however, to have heterozygote *excess* (see Table 2). HLA class I genes have lower read depth. The HLA class II genes map into and after the third and largest HW spike in the MHC region. Most of the class II genes are characterized by positive inbreeding coefficients and a severe deficiency of heterozygotes (see Table 2). We found 29 variants in the class II region that had no heterozygotes, but were clearly polymorphic (MAF $\ge$0.05), which is highly unlikely under HWE. Class II genes DQA1, DQB1 and DRB1 have lower read depth.

Some of the spikes in the MHC region consist of variants occurring inside segmental duplications, but there are also several spikes of significant variants outside such duplications.

**Table 2:** Descriptive statistics for certain genomic areas of the JPT sample: number of SNPs, percentage monomorphic markers, percentage significant markers in a HW exact test with $\alpha = 0.001$, percentage of significant markers due to heterozygote excess and medians of the inbreeding coefficient (*f*) and the median read depth (DP)

	#SNPs	%Mono	%Sig	%HetExc	Med (*f*)	Med (DP)
CHR 6	5,008,031	83.87	1.26	22.29	−0.005	17,811
MHC	136,176	59.56	11.86	1.38	−0.005	16,917
Outside MHC	4,871,855	84.55	0.49	59.58	−0.006	17,854
HLA-A	348	37.36	1.83	50.00	−0.005	15,622
HLA-B	308	37.99	2.62	60.00	−0.002	10,426
HLA-C	329	44.68	5.49	90.00	0.016	13,691
HLA-DPA1	943	54.29	0.46	0.00	0.207	18,285
HLA-DPB1	763	44.95	6.19	0.00	0.142	17,937
HLA-DQA1	869	9.21	22.69	0.00	0.121	11,898
HLA-DQB1	905	6.52	30.14	0.00	0.142	10,915
HLA-DRB1	837	8.96	26.12	5.53	0.135	11,396
HLA-DRA	162	54.32	0.00	0.00	−0.010	19,879
CHR 21	1,102,563	84.54	1.62	80.06	−0.005	17,734
CHR 21 p.arm	23,453	85.57	36.04	85.25	−0.035	40,683
CHR 21 q.arm	1,072,796	84.54	0.56	60.02	−0.005	17,662
CHR 1-(Cen)	16,761	0.00	9.67	82.60	−0.010	19,402
CHR 1-outside	6,434,666	85.19	0.54	73.41	−0.007	18,017
CHR 2-(Cen)	8962	0.00	4.71	84.83	−0.005	18,816
CHR 2-outside	7,054,536	85.61	0.45	61.97	−0.005	17,948
CHR 3-(Cen)	17,114	0.00	0.62	94.34	−0.005	17,224
CHR 3-outside	5,800,292	85.23	0.48	53.68	−0.006	17,959
CHR 4-(Cen)	9621	0.00	4.77	89.98	−0.005	19,050
CHR 4-outside	5,708,252	84.88	0.49	53.58	−0.006	17,509
CHR 4 (4pTel)	4734	87.11	8.03	100.00	−0.020	18,861
CHR 10 (10pTel)	3704	87.53	5.19	0.00	−0.012	17,610
CHR 12 (12pTel)	2163	85.71	24.92	100.00	−0.020	28,209
CHR 14 (14pTel)	11,094	91.47	8.56	64.20	−0.005	22,173
Autosomes	80,931,043	85.01	0.62	58.93	−0.007	17,889

We repeated the analysis for another sample in order to see if these MHC spikes were specific for the JPT sample. Supplementary Figure S7 shows the HW track for the same area for the Yoruba sample (YRI), and reveals a similar picture, with three composite spikes in the MHC region with mainly heterozygote deficiency.

### The p-arms of acrocentric chromosomes 21 and 22

**Fig. 4:**
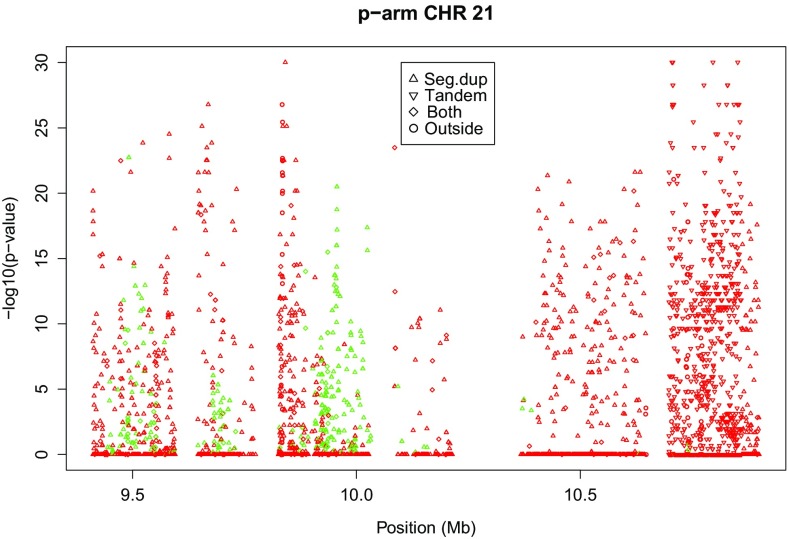
Plots of exact *p* values on the p-arm of chromosome 21 (*green* $f > 0$, *red* $f < 0$)

Chromosome 21 has a p-arm of 11 Mb, and this whole arm is an area with a very high rate of disequilibrium (36% significant, see Table 2). If the HW analysis is stratified according to the two arms, then the q arm has 0.56% significant variants, a rate that is comparable to that of most autosomes. Chromosome 22, the smallest autosome, also has a relatively high disequilibrium rate towards 22pTel. However, 22 has no variants with RS identifier and less than 5% missings on its p-arm. Figure 4 shows the p-arm of chromosome 21. Symbols indicate if a variant is situated inside a segmental duplication, inside a simple tandem repeat, inside both or outside. This shows that towards 21pTel almost all variants occur in segmental duplications. Towards the centromere, many variants occur in tandem repeats. The p-arm of 21 is characterized in general by strong heterozygote excess, though there are some areas with heterozygote deficiency too.We note that this p-arm has extremely high read depth.

### Centromeres

**Fig. 5:**
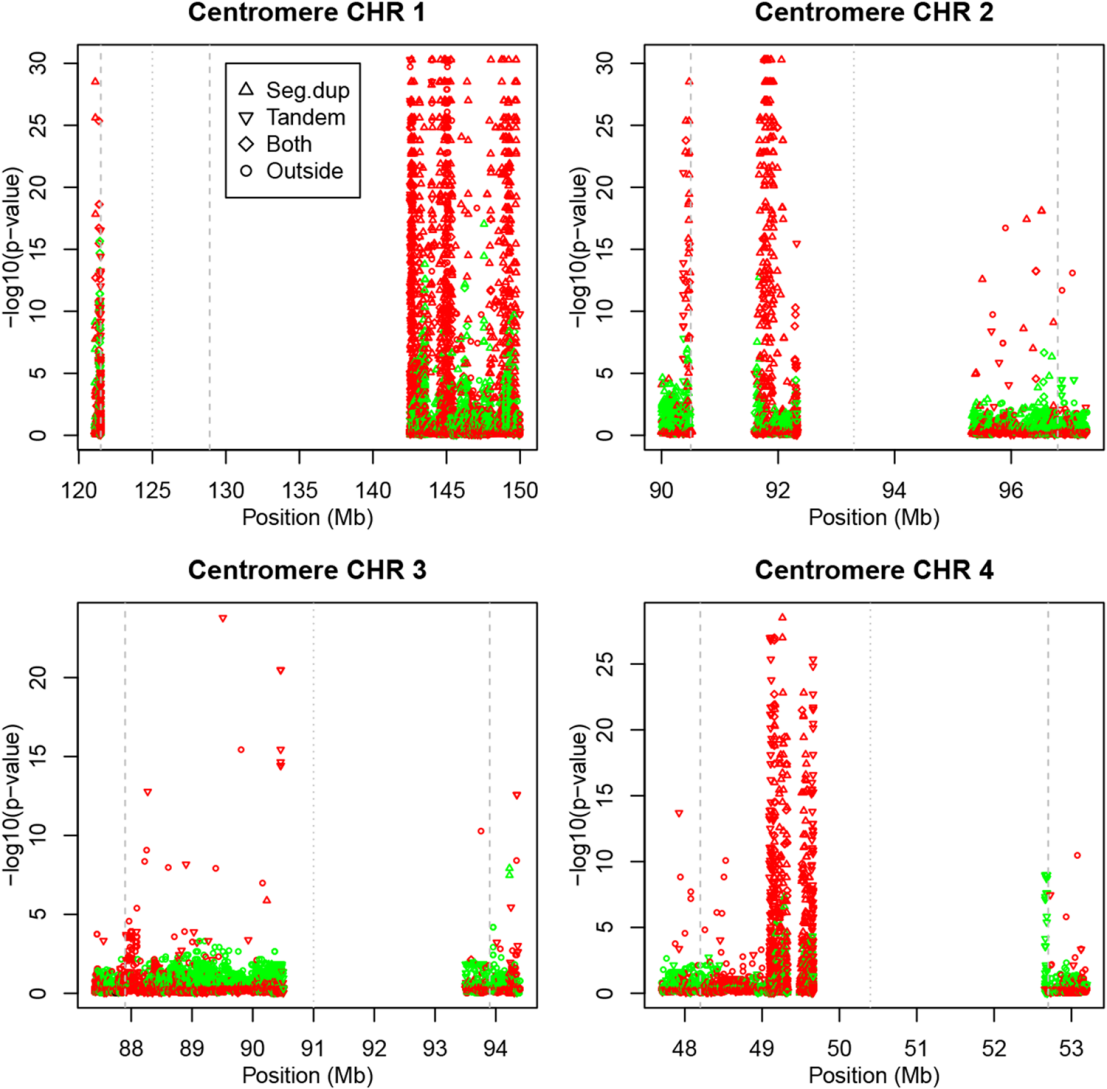
Plots of exact *p* values around the centromeres of chromosomes 1–4 (*green* $f > 0$, *red* $f < 0$). *Vertical lines* indicate limits and center of the centromere

Most chromosomes, though not all, show high rates of HWD in the regions that flank the centromere. HW tracks of the areas flanking the centromeres for the first four chromosomes are shown in Fig. 5. We used the documented limits of the centromere for build hg19 (GRCh37) plus an extra margin of half a megabase before and after the centromere. For chromosome 1, the documented limit on the q arm of the centromere was extended with a distal 21.1Mb in order to reveal the spike.

For chromosomes 2, 3 and 4 many variants are seen to fall within the centromere region. Chromosomes 2 and 4 have strong HWD spikes with heterozygote excess inside the centromere. Chromosomes 1 and 2 have HWD spikes with heterozygote excess just on the p-arm, proximal to the centromere region. Figure 5 shows that the significant variants inside and flanking the centromeres consist almost exclusively of variants pertaining to segmental duplications and simple tandem repeats. The statistics in Table 2 show that the centromere regions do not contain monomorphic markers, and have, with the exception of chromosome 3, a rate of significant variants that is about 10 times higher or more in comparison with the rest of the chromosome. Read depth tends to be higher in the centromere region.

### Telomeres

**Fig. 6:**
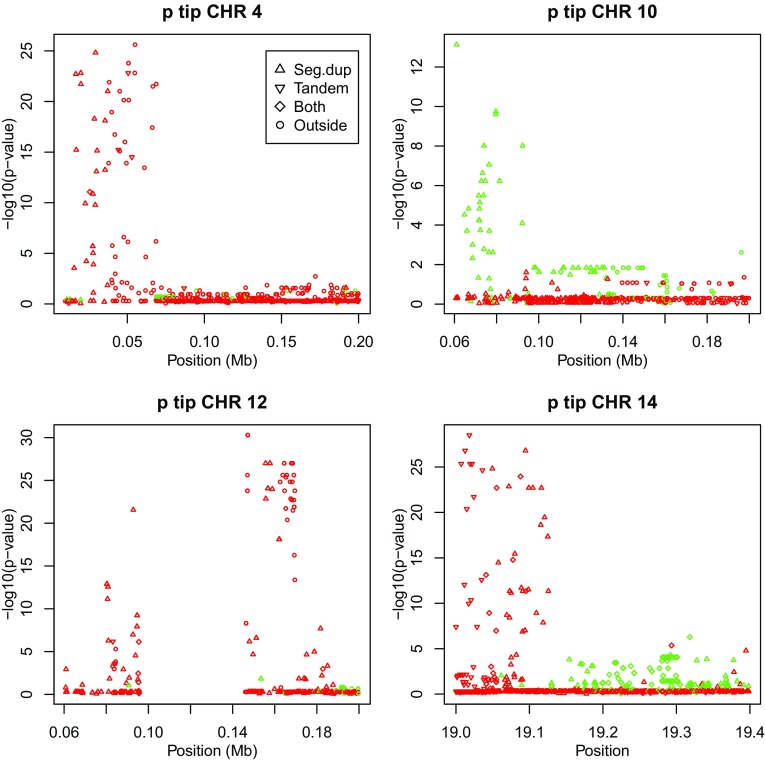
Plots of exact *p* values at the p tip of chromosomes 4, 10, 12 and 14 (*green* $f > 0$, *red* $f < 0$)

Several chromosomes show a disequilibrium spike close to the telomere at the tip of their p-arm. This is shown in Fig. 6 for chromosomes 4, 10, 12 and 14. For chromosomes 4, 10, and 12 we show the first 0.2 Mb, whereas for chromosome 14 the first 19.4 Mb were needed to reveal the spike at the tip of the p-arm. Three of the four tips shown have strong heterozygote excess as shown by the statistics in Table 2.

Figure 6 shows that for chromosomes 4 and 10 the distal spikes on the p-arm consists of variants occurring in segmental duplications, and for chromosome 14 the p-distal spike has variants in tandem repeats followed by variants in segmental duplications. Chromosomes 4 and 12 also have sets of significant markers proximal of the area with segmental duplications.

### X-chromosome

**Fig. 7:**
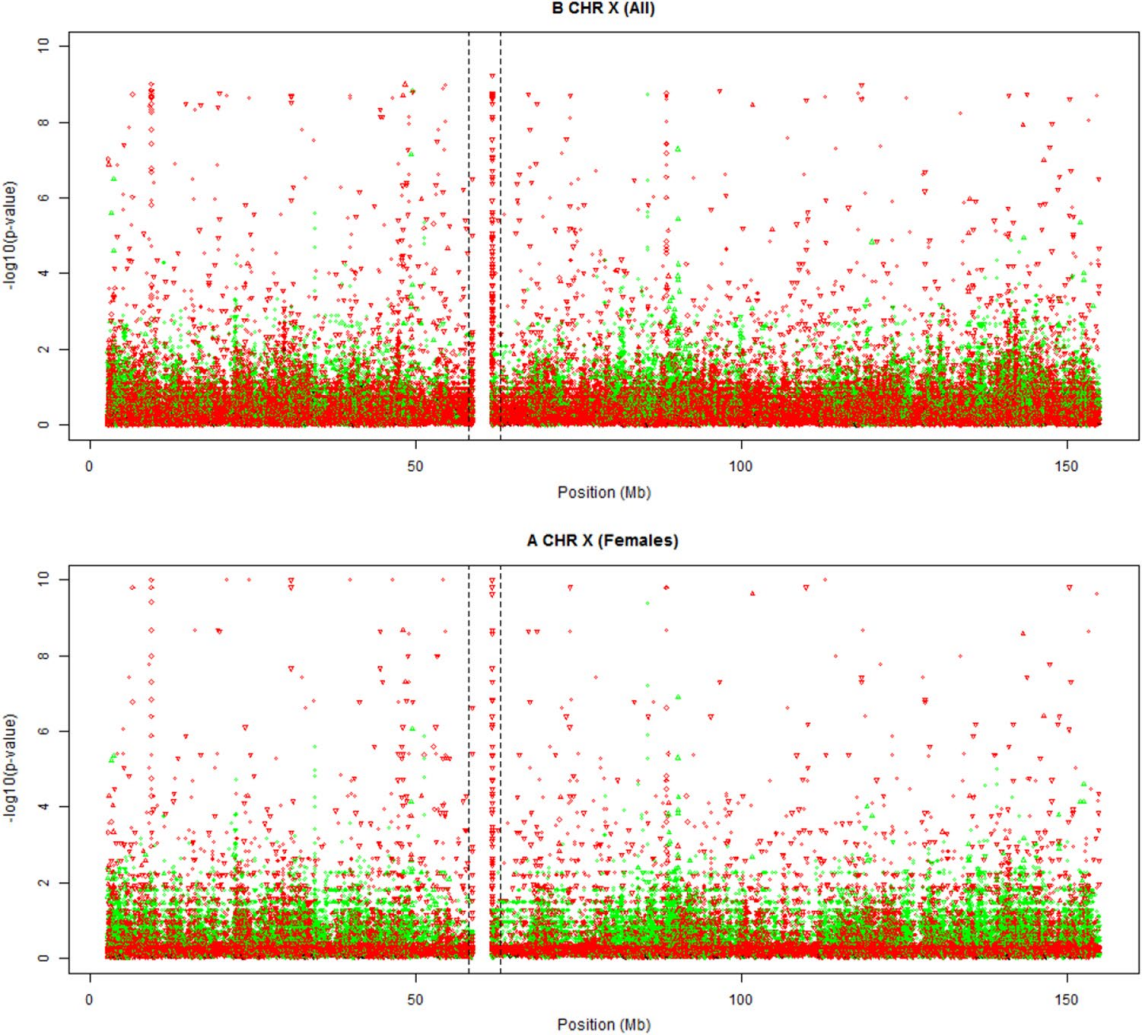
Plots of exact mid *p* values of chromosome X. **a** Testing females only. **b** Testing males and females (*green* $f > 0$, *red* $f < 0$). *Dashed vertical lines* indicate the limits of the centromere region

The X-chromosome is as large as chromosome 7, but has fewer variants with RS identifiers. We show the full track of the X-chromosome in Fig. 7, using the females-only and all-individuals exact test. Both plots reveal a HWD spike inside the centromere, and some incidental spikes. PAR regions are not shown because of a lack of RS markers in those regions. The all-individuals test shows more significant results because it is also sensitive to differences in allele frequencies between the sexes.

The disequilibrium spike inside the centromere corresponds to variants occurring inside simple tandem repeats. The track also reveals some additional spikes, at 9.370–9.385 Mb and at 88.455–88.465 Mb, with variants pertaining to both segmental duplications and simple tandem repeats. The latter spike falls inside the hypothesized PAR3 region (Veerappa et al. 2013).

### Horizontal bands of *p* values

**Fig. 8:**
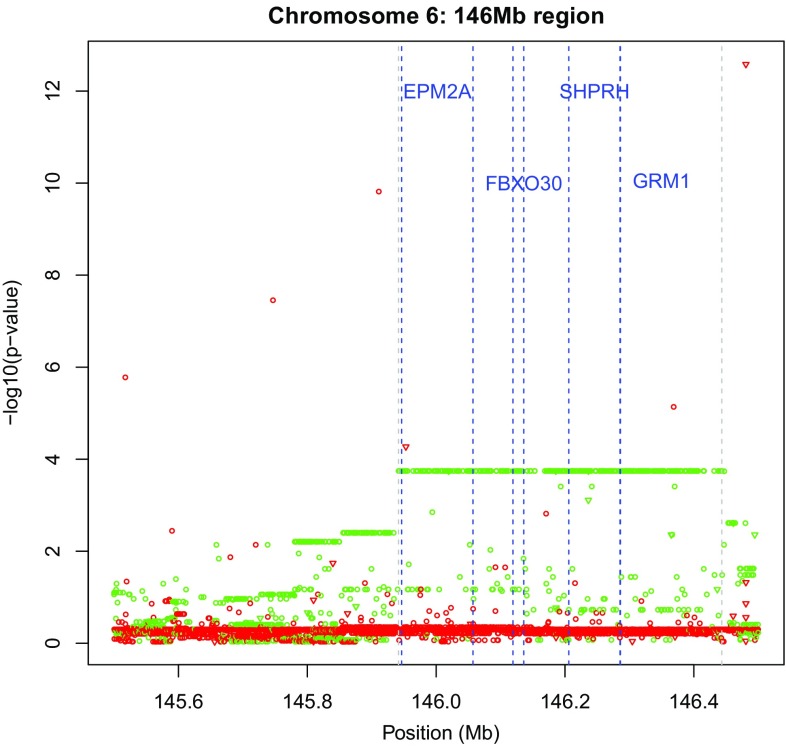
Plots of exact *p* values at 145.5–146.5 Mb on chromosome 6 (*green* $f > 0$, *red* $f < 0$). Monomorphic markers not shown

In some HW tracks horizontal bands of *p* values were observed. This was particularly manifest in the area around 146 Mb on the q arm of chromosome 6. The horizontal bands imply that there are many variants that have exactly the same genotypic composition. This phenomenon is shown in Fig. 8 where the largest green horizontal line refers to variants that all have the same genotypic composition (AA = 97, AB = 4, BB = 3), which has a deficiency of heterozygotes. The horizontal line comprised 329 variants with this genotypic composition, and all 104 individuals are identical with respect to the 329 variants. These variants are not contiguous but are interspersed with other variants that are mostly monomorphic. The band (145,941,639–146,443,329) spans an area of 0.5 Mb containing 1702 variants (monomorphics excluded) in total. If the individuals are phased for the 329 variants using R package haplo.stats (Sinnwell and Schaid 2016), then there exist only two haplotypes that unambiguously explain the genotype data, having probabilities that equal the allele frequencies of all involved markers. The band covers the genes EPM2A (laforin glucan phosphatase), FBXO30 (F-box protein 30), SHPRH (histone linker phd ring helicase) and part of GRM1 (glutamate metabotropic receptor 1).

Two shorter adjacent stripes with heterozygote deficiency are observed between 145.7 and 146 Mb, with compositions (AA = 92, AB = 9, BB = 3) and (AA = 91, AB = 10, BB = 3). The variants of these stripes are not contiguous, as they are interrupted by markers that have a different genotypic composition. The two shorter stripes can be interpreted as pertaining to the same haplotype as the longest stripe, but having, respectively, 5 and 6 AA homozygotes recorded as heterozygotes. Inspection of the data shows that the shorter stripes also consist of only two haplotypes that again unambiguously explain the genotype data. Each stripe has therefore three genotypes. When the three stripes are combined, five different genotypes are found. Moreover, three long red stripes with non-significant *p* values with heterozygote excess are also observed. Due to the presence of many low MAF markers in the data base, these stripes are less surprising, as they correspond to relatively common patterns (e.g., AA = 103, AB = 1, BB= 0).

### Incidental spikes

**Fig. 9:**
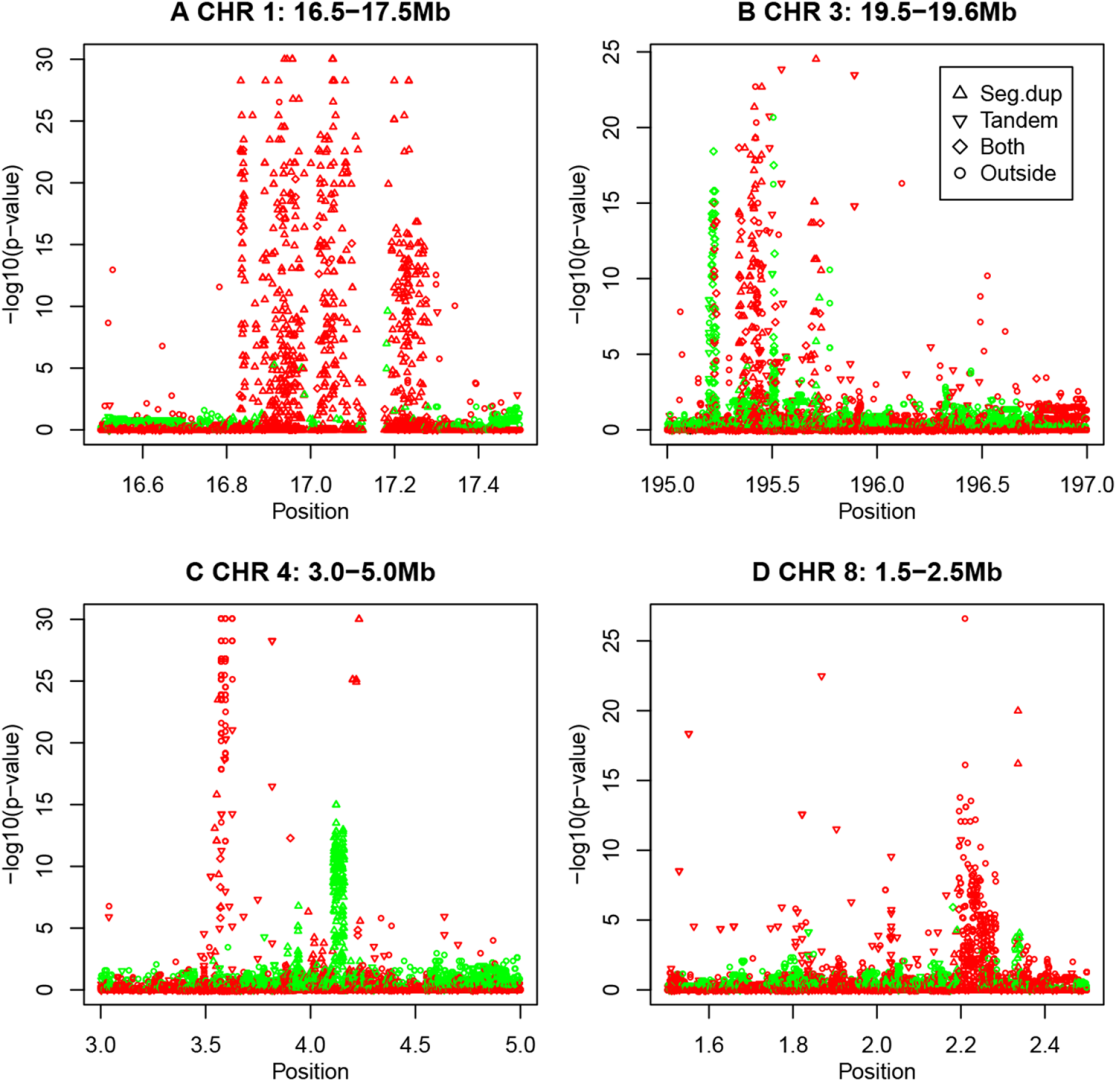
Spikes of HWE exact *p* values on four different chromosomes (*green* $f > 0$, *red* $f < 0$)

Finally, many incidental spikes of strong HWD are observed outside the aforementioned areas in the preceding sections. A few salient spikes are shown in Fig. 9.

This figure shows that spikes are of a homogeneous nature in the sense that each individual spike is characterized by having all its variants with excess or deficiency of heterozygotes. The 17 Mb spike on chromosome 1 concerns variants inside segmental duplications, almost universally with heterozygote excess. The spikes on chromosome 3 have variants in duplications and in tandem repeats. Chromosome 4 and 8 show spikes of heterozygote excess that do not coincide with duplications or repeats. Chromosome 4 shows a duplication almost exclusively characterized by deficiency of heterozygotes.

### Relation with read depth

**Fig. 10:**
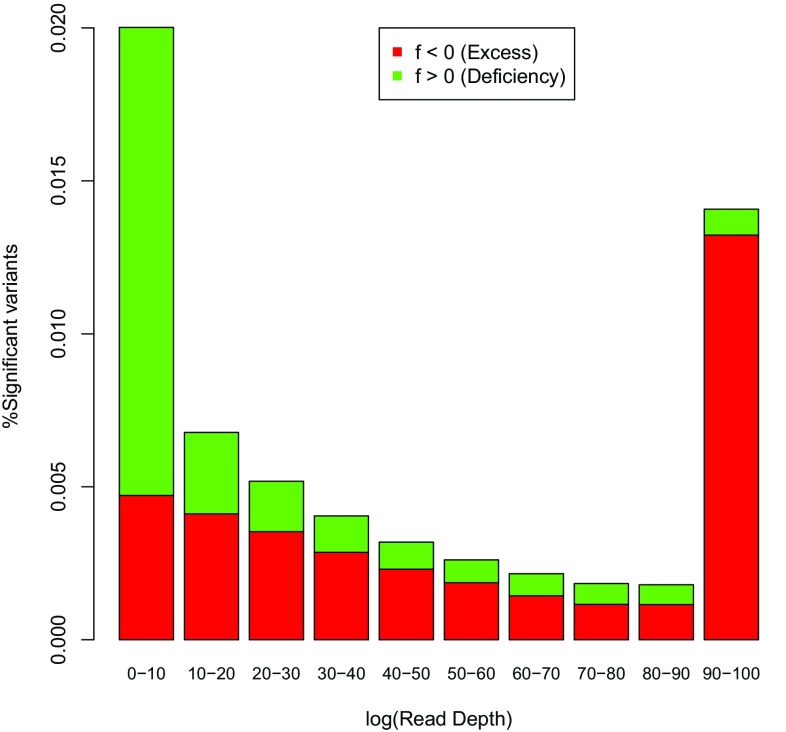
Percentage of significant HWD as a function of read depth (*green* $f > 0$, *red* $f < 0$)

Read depth is recorded in phase 3 of the 1000 genomes project data files as the total read depth per variant, that is, summed over the 104 individuals. Figure 10 shows the percentage of significant variants with excess and deficiency of heterozygotes as a function of the read depth (DP) decile, using all autosomal polymorphic variants that have less than 5% missing values. The figure shows that more disequilibrium is found in the tails of the read depth distribution. The rate of significant markers goes down with increasing read depth, up to a limit. Extremely high read depth brings about more HWD. The figure also shows that extremely high read depths are, in general, associated with an excess of heterozygotes, and read depths below the median are more often associated with a deficiency of heterozygotes.

### Relation with segmental duplications and with simple repeat regions

**Fig. 11:**
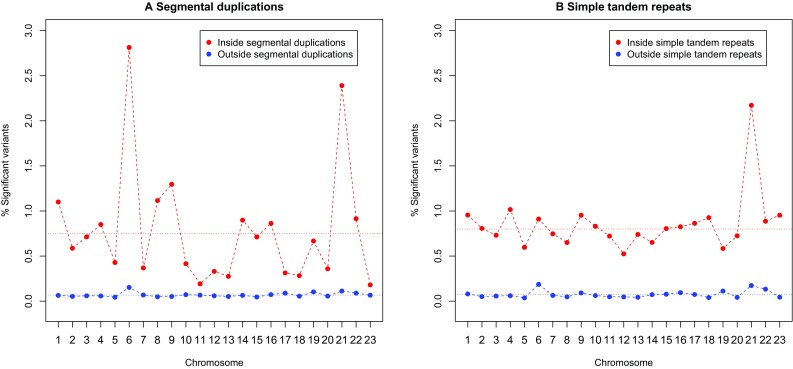
**a** Percentage of significant HWD in and outside segmental duplications for each chromosome. **b** Percentage of significant HWD in and outside simple tandem repeats for each chromosome. *Dotted horizontal lines* represent the overall autosomal rate

We stratified the results of our exact tests for HWP according to whether variants occurred in areas with segmental duplications or not, using the annotation on segmental duplications of at least 1Kb in the UCSC genome browser (Bailey et al. 2002). Figure 11a shows the percentage of significant variants per chromosome, stratified for segmental duplications. The overall rate of significant variants inside duplications (0.751%) is about 11 times higher as the rate outside duplications (0.068%).

Chromosome 6 (with the MHC region) and chromosome 21 (with a lot of HWD in its p-arm) are outlying with many significant variants in segmental duplications. Likewise, exact test results were also stratified according to inclusion in simple tandem repeats, using the simple tandem repeat tracks of the UCSC genome browser (Benson 1999). Figure 11b shows the percentage of significant variants per chromosome, stratified for simple repeats. The overall rate of significant variants inside simple repeats (0.800%) is about 11 times higher as the rate outside simple repeats (0.072%).

Because of the higher rate of HWD in repeats and duplications, we recalculated the descriptive statistics in Table 1 excluding all variants in segmental duplications and simple tandem repeats, to obtain the results in Supplementary Table S1. The overall rate of significant variants decreases from 0.6 to 0.3%, and the rate of HWD on each chromosome about halved. However, the rate still is three times higher as expected by chance alone, and about 56% of the remaining disequilibrium is still due to heterozygote excess.

**Fig. 12:**
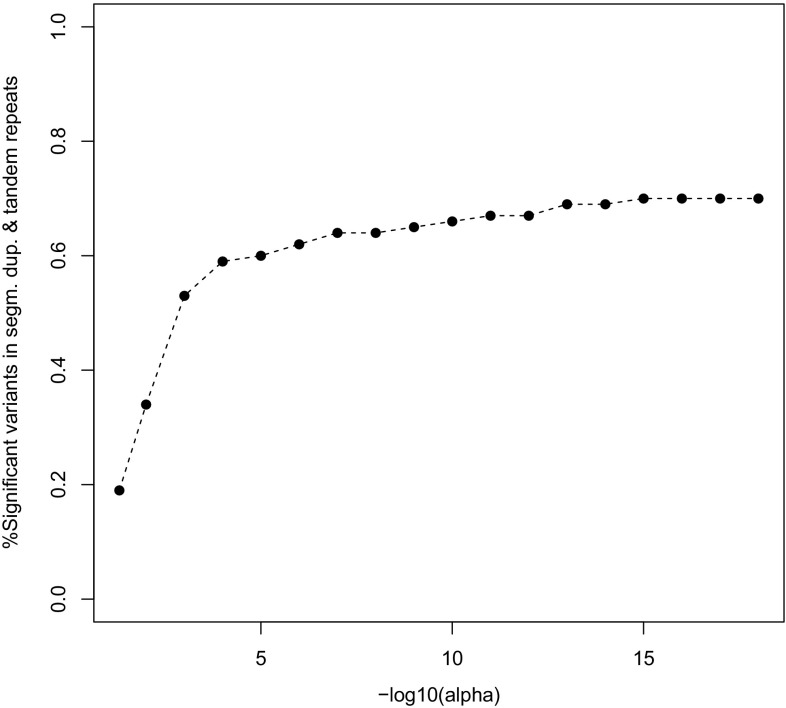
Percentage of significant HWD in and outside segmental duplications or tandem repeat regions for each chromosome as a function of the significance threshold ($\alpha$)

The JPT genome studied in this paper has 3.8% of all of its variants in segmental duplications, and 3.0% in simple tandem repeat regions, totaling an overall of 6.8% variants in areas with copy number variation. If we focus on the variants with significant HWD, then over 60% of these is found in areas with segmental duplications or simple tandem repeats. Figure 12 shows the percentage of significant variants in these areas as a function of the significance threshold ($\alpha$).

According to Fig. 12, at the HapMap exclusion level of $\alpha =0.001$, 59% of the significant variants is inside segmental duplications or simple tandem repeat regions, and this monotonically increases if $\alpha$ is made smaller, showing how copy number variation accumulates in the tail of the *p* value distribution. The top ten most significant variants of each chromosome almost invariably consist of variants with 100% heterozygosity that occur predominantly inside segmental duplications or simple tandem repeats.

The analysis described in this section was repeated for 107 individuals of the Yoruba (YRI) sample of the 1000 Genomes project. This sample consists mainly of parent–offspring trios, and children were eliminated from the database prior to analysis. Of some existing sibling pairs, one individual was removed too, in order to best satisfy the assumption of a sample of unrelated individuals. All corresponding graphics and tables for the YRI sample are included as supplementary material in Appendix B. The YRI sample clearly shows less significant HWD than the JPT sample as shown in Figure S5. The rate of significant markers is about 50% lower for the YRI sample, and also increased as a function of the percentage of missings. The YRI sample has about 13% less monomorphic variants on the autosomes, and about 23% less monomorphic variants on the X-chromosome. The median inbreeding coefficient is the same for all chromosomes, which can be ascribed to the existence of many low MAF variants on all chromosomes. HWD spikes for the MHC region, p-arm of chromosome 21, centromeres, telomeres were also observed in the YRI sample. Reported incidental spikes for the JPT sample were also observed in the YRI sample. The horizontal band corresponding to a haplotype on chromosome 6 at 146 Mb was not observed in the YRI sample. Similar associations between HWD and read depth and HWD and segmental duplications and tandem repeat regions were also found for the YRI sample.

## Discussion and conclusion

Calculations in this paper show there is more significant HWD in the genome than is expected to occur by chance alone under the assumption of independent markers. Disequilibrium is found to be most often due to heterozygote excess. Disequilibrium rates are 11 times larger in simple tandem repeat regions and in segmental duplications, suggesting HWD to be due to, at least in part, sequencing problems that arise from the existence of multiple copies of a polymorphism. This would explain why increased HWD is more often observed in genomic regions where duplications (MHC region) and repetitive DNA sequences (centromeres) are known to occur. In the following we discuss causes that can generate higher rates of HWD. We suggest heterozygote lack to be due to null alleles, and heterozygote excess to be due to duplication.

Null alleles can arise from substitutions and indel mutations (Crooks et al. 2013). An individual having one normal allele and a null allele at a locus, is easily misclassified as a homozygote. This causes bias in the estimation of the allele frequencies and artificially inflates the degree of homozygosity. If the number of null alleles is substantial, a test for HWP may indicate significant lack of heterozygotes, whereas in reality the locus has more than two alleles. Heterozygote individuals carrying null alleles can be expected to produce fewer aligned reads, and homozygous null individuals can be expected to have no aligned reads at all, producing missing values. This way, null alleles can provoke smaller read depth and deficiency of heterozygotes.

We hypothesize that duplication is a mechanism that generates heterozygote excess. Let the variant that is assayed be a *G*/*T* polymorphism, which is duplicated together with its flanking sequences. In theory, such a duplication could be present in the reference genome or in the sampled individuals, or in both. Here we treat the reference genome as being unique, though the same consequences can be expected for a duplication that occurs in the reference genome. We distinguish original and duplication by indicating the genotypes of the original polymorphism by $G_1G_1$, $G_1T_1$ and $T_1T_1$, and those of its duplicate by $G_2G_2$, $G_2T_2$ and $T_2T_2$. Assuming both copies to be reasonably polymorphic (eventually having the same or similar allele frequencies) implies that double homozygote genotypes $G_1G_1T_2T_2$ and $T_1T_1G_2G_2$ exist but these will be typed as heterozygotes because these individuals carry both the *T* allele and the *G* allele. Additionally, single homozygote genotypes like $T_1T_1T_2G_2$ can also be typed as heterozygotes while in fact they are homozygous at the original locus. The confusion of both polymorphisms by the genotyping technology will give an increased heterozygosity.

In the most extreme case, the two polymorphisms may be fixed for different alleles, e.g., all individuals being $T_1T_1G_2G_2$. Such a situation could arise if, in the course of evolution, a duplication arises but with a copying error at the assayed nucleotide, or a duplication is followed by a point mutation at the interrogated base. In this case all individuals will carry both alleles and the heterozygosity is 100%. For a single, unduplicated bi-allelic marker, observing 100% heterozygosity is highly unlikely under HWE, and only possible if the marker is maximally polymorphic with allele frequency 0.5. Genotyping results will suggest under these circumstance one variant with maximal and significant HWD, whereas the underlying two loci are in fact monomorphic and in truth cannot contradict HWE. The NGS data of the JPT populations suggests this indeed occurs, as we found 424 variants to consist of heterozygotes only.

There are also markers with an extremely high number of heterozygotes, but less than 100% heterozygosity. This can be explained by having one polymorphism fixed ($T_1T_1$) and the second polymorphism being a $T_2/G_2$ polymorphism with low MAF for the $T_2$ allele. Most genotypes will be $T_1T_1G_2G_2$ which will all be typed as heterozygotes, some will be $T_1T_1T_2G_2$ (assayed as heterozygotes) and there will be almost no $T_1T_1T_2T_2$ individuals (0 or close to zero count). Again, observed heterozygosities will be lifted. Evidently, more complicated patterns can arise if a sequence including a variant is not duplicated once, but several times.

Areas with a high read depth (many aligned reads) suggest there exists copy number variation for the interrogated locus in the sampled individuals, and such areas are indeed characterized by heterozygote excess (see Fig. 10). This fortifies our argument that duplications are indeed responsible  for the observed heterozygote excess.

The other extreme of the spectrum, having no heterozygotes for polymorphic markers with an MAF above 0.05, was also found in the JPT data for 88 autosomal variants. Most of these were in the class II genes of the MHC region, and at a HWD spike on chromosome 4 between 69.38–69.49 Mb.

Excess disequilibrium can, at least in part, also be due to LD. If many variants reside on a haplotype, and the haplotype locus is out of HWP, then many of its constituent variants can be expected to be out of HWP too. The horizontal band in Fig. 8 is in fact an example of this phenomenon. However, if a haplotype locus is in agreement with HWP, one can expect all its variants to be in agreement too. The presence of LD implies that the inbreeding coefficients of contiguous variants are similar (Weir et al. 2004). We do however, not expect LD to specifically generate heterozygote excess.

The HW analysis of the YRI data included in the appendix shows that this sample has relatively fewer significant variants. We note that the rates of significant variants of the two samples cannot directly be compared, even though the two samples have approximately the same size. The YRI sample has more variants, and relatively many more rare variants with a low MAF. Statistical tests for HWP with rare variants have low power (Emigh 1980; Wigginton et al. 2005; Graffelman and Moreno 2013) and thus there is less power to detect HWD in the African sample, and it is thus unsurprising that fewer significant results are observed in the YRI sample. Because the distribution of the minor allele frequency is different in each sample, the rates of significant variants are incommensurable.

In gene–disease association studies variants are sometimes excluded on the basis of their HW *p* value prior to association analysis, in order to avoid genotyping error. Copy number variation is known to play an important role in genetic disease (Beckmann et al. 2007). Our results suggest such exclusion is not be recommended: significant HW *p* values are potential indicators of the existence of copy number variation, and by blind filtering on the HW *p* value, one might precisely be filtering out the disease-related genetic factors.

We found it noticeable that NGS data are in general, characterized by heterozygote excess. With SNP array data, the situation is precisely the reverse: heterozygote deficiency is more common than heterozygote excess, which can be explained by the existence of null alleles (Graffelman et al. 2015). Our final conclusion is that sequence duplication is a main factor producing Hardy–Weinberg disequilibrium. HW tests can also detect long-range haplotypes, and uncover genomic areas in disequilibrium for other reasons. Tests for Hardy–Weinberg proportions remain an invaluable tool for the analysis and quality control of next generation sequence data.

## Electronic supplementary material

Below is the link to the electronic supplementary material.

Supplementary material 1 (PDF 2046 KB)

## References

[CR1] Bailey JA, Gu Z, Clark RA, Reinert K, Samonte RV, Schwartz S, Adams MD, Myers EW, Li PW, Eichler EE (2002). Recent segmental duplications in the human genome. Science.

[CR2] Beckmann JS, Estivill X, Antonarakis SE (2007). Copy number variants and genetic traits: closer to the resolution of phenotypic to genotypic variability. Nature Rev Genet.

[CR3] Benson G (1999). Tandem repeats finder: a program to analyze DNA sequences. Nucl. Acids Res..

[CR4] Crooks L, Carlborg O, Marklund S, Johansson AM (2013). Identification of null alleles and deletions from SNP genotypes for an intercross between domestic and wild chickens. G3 (Bethesda).

[CR5] Crow JF, Kimura M (1970). An introduction to population genetics theory.

[CR6] Emigh TH (1980). A comparison of tests for Hardy–Weinberg equilibrium. Biometrics.

[CR7] Gomes I, Collins A, Lonjou C, Thomas NS, Wilkinson J, Watson J, Morton N (1999). Hardy–Weinberg quality control. Ann Human Genet.

[CR8] Graffelman J, Moreno V (2013). The mid $$p$$-value in exact tests for Hardy–Weinberg equilibrium. Stat Appl Genet Mol Biol.

[CR9] Graffelman J, Nelson SC, Gogarten SM, Weir BS (2015). Exact inference for Hardy–Weinberg proportions with missing genotypes: single and multiple imputation. G3 (Genes, Genom, Genet).

[CR10] Graffelman J, Weir BS (2016). Testing for Hardy–Weinberg equilibrium at bi-allelic genetic markers on the X chromosome. Heredity.

[CR11] Graffelman J (2015) Exploring diallelelic genetic markers: the Hardy–Weinberg package. J Stat Softw 64(3):1–23. http://www.jstatsoft.org/v64/i03/

[CR12] Hartl DL (1980). Principles of population genetics.

[CR13] Hosking L, Lumsden S, Lewis K, Yeo A, McCarthy L, Bansal A, Riley J, Purvis I, Xu C (2004). Detection of genotyping errors by Hardy–Weinberg equilibrium testing. Eur J Human Genet.

[CR14] Leal SM (2005). Detection of genotyping errors and pseudo-SNPs via deviations from Hardy–Weinberg equilibrium. Genet Epidemiol.

[CR15] Nielsen R, Joshua SP, Albrechtsen A, Song YS (2011). Genotype and SNP calling from next-generation sequencing data. Nat Rev Genet.

[CR16] Purcell S, Neale B, Todd-Brown K, Thomas L, Ferreira MAR, Bender D, Maller J, Sklar P, de Bakker PIW, Daly MJ, Sham PC (2007) PLINK: a toolset for whole-genome association and population-based linkage analysis. Am J Human Genet 81(3):559–575. http://pngu.mgh.harvard.edu/purcell/plink/10.1086/519795 PMC1950838 17701901

[CR17] Rohlfs RV, Weir BS (2008). Distributions of Hardy–Weinberg equilibrium test statistics. Genetics.

[CR18] Sinnwell JP, Schaid DJ (2016) Haplo.stats: statistical analysis of haplotypes with traits and covariates when linkage phase is ambiguous. R package version 1.7.7. https://CRAN.R-project.org/package=haplo.stats

[CR19] Teo YY, Fry AE, Clark TG, Tai ES, Seielstad M (2007). On the usage of HWE for identifying genotyping errors. Ann Human Genet.

[CR20] The International HapMap Consortium (2007) A second generation human haplotype map of over 3.1 million SNPs. Nature 449(7164):851–861 10.1038/nature06258 PMC2689609 17943122

[CR21] The MHC sequencing consortium (1999). Complete sequence and gene map of a human major histocompatibility complex. Nature.

[CR22] The 1000 Genomes Project Consortium (2015) A global reference for human genetic variation. Nature 526:68–74 10.1038/nature15393 PMC4750478 26432245

[CR23] Traherne JA (2008). Human MHC architecture and evolution: implications for disease association studies. Int J Immunogenet.

[CR24] Veerappa AM, Padakannaya P, Ramachandra NB (2013). Copy number variation-based polymorphism in a new pseudoautosomal region 3 (PAR3) of a human X-chromosome-transposed region (XTR) in the Y chromosome. Funct Integrat Genom.

[CR001] Weir BS (1996) Genetic Data Analysis II. Sinauer Associates, Massachusetts

[CR25] Weir BS, Hill WG, Cardon LR (2004). Allelic association patterns for a dense SNP map. Genet Epidemiol.

[CR26] Wigginton JE, Cutler DJ, Abecasis GR (2005). A note on exact tests of Hardy–Weinberg equilibrium. Am J Human Genet.

