# Supplementary material for: A genome-wide study of Hardy–Weinberg equilibrium with next generation sequence data
Source: Hum Genet. 2017 Apr 3;136(6):727–41. doi: 10.1007/s00439-017-1786-7 (PMC5429372; doi:10.1007/s00439-017-1786-7)
Supplement: Supplementary file 1 — Supplementary material 1 (PDF 2046 KB) [file 439_2017_1786_MOESM1_ESM.pdf]

Appendices

A Appendix: Supplementary material for the JPT sample

A.1 Exact p-value as a function of MAF

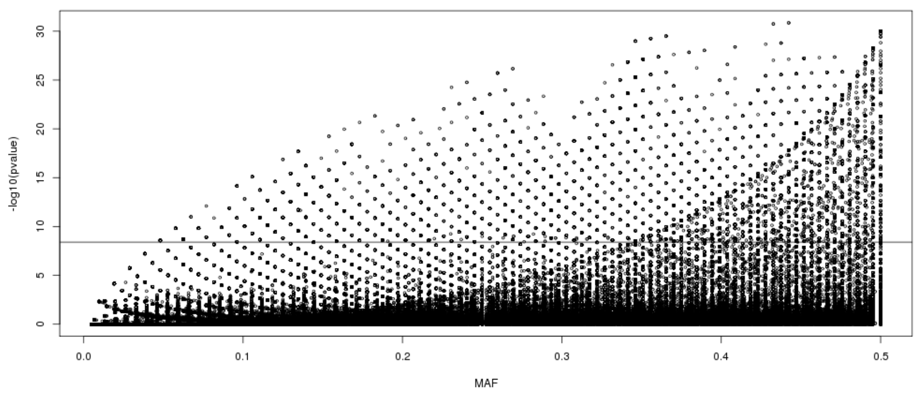

Figure S1: Supplementary Figure. Exact HW p-value as a function of the minor allele frequency (MAF) for 12.4 million polymorphic variants of the JPT sample.

A.2 QQ-plots of Exact p-values for each chromosome

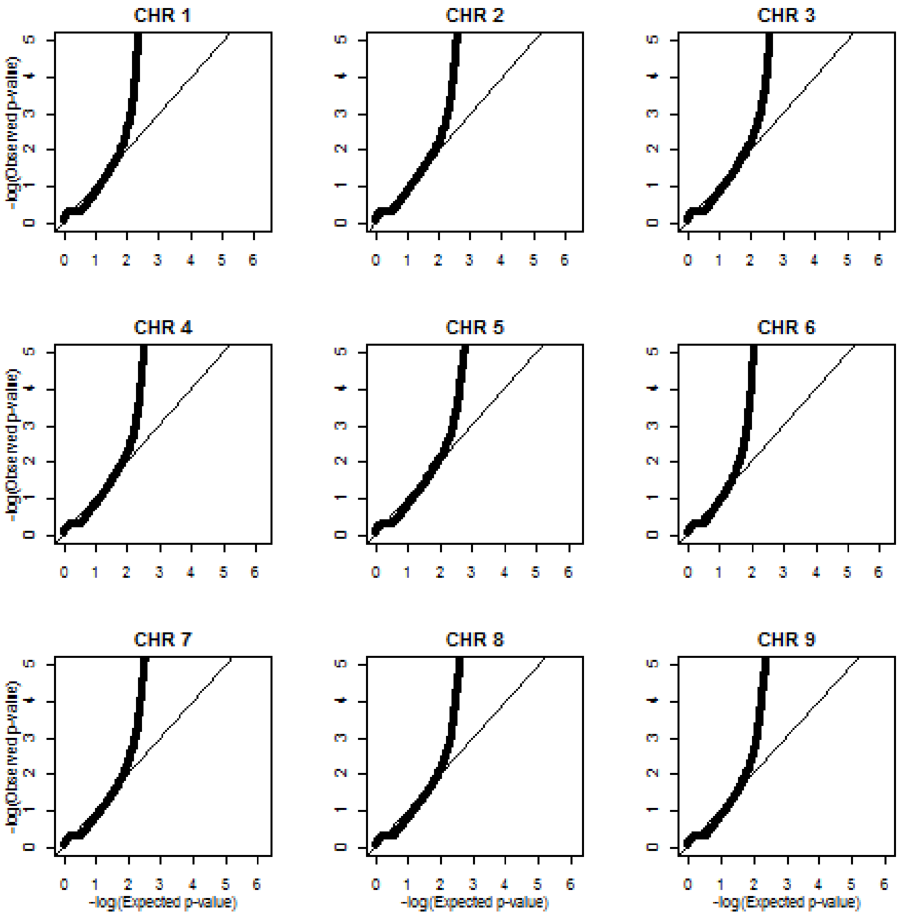

Figure S2: Supplementary Figure. QQ-plots of Exact HW p-values for chromosomes 1 through 9 against a uniform distribution.

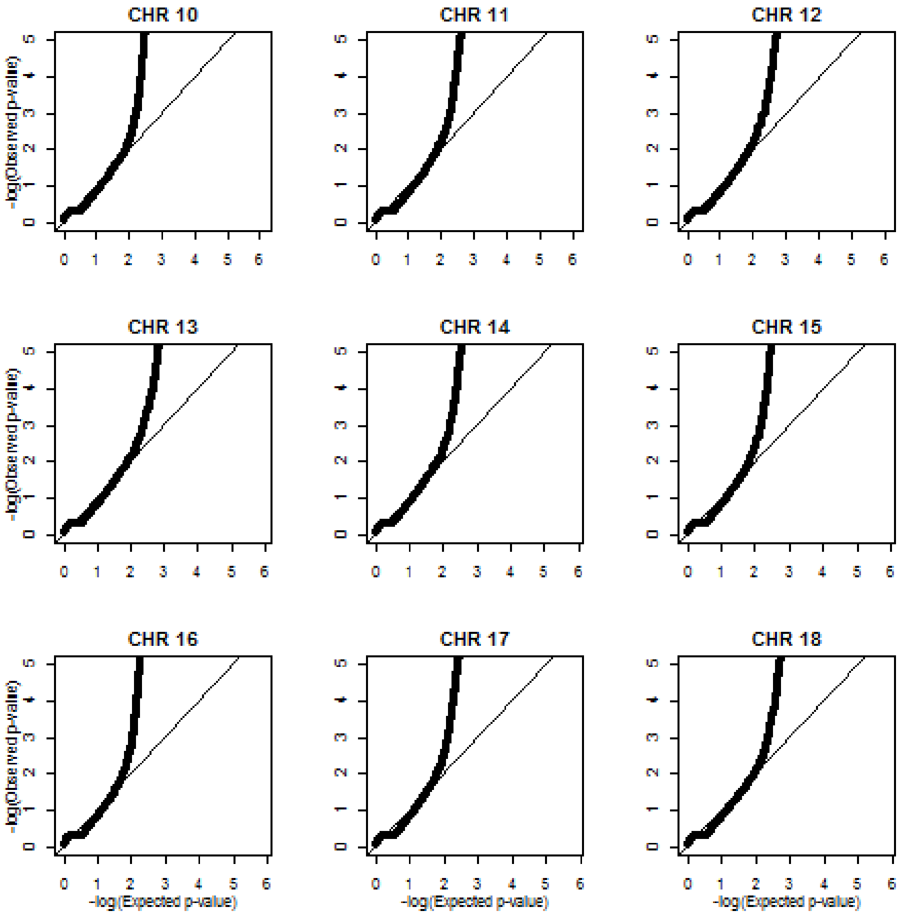

Figure S3: Supplementary Figure. QQ-plots of Exact HW p-values for chromosomes 10 through 18 against a uniform distribution.

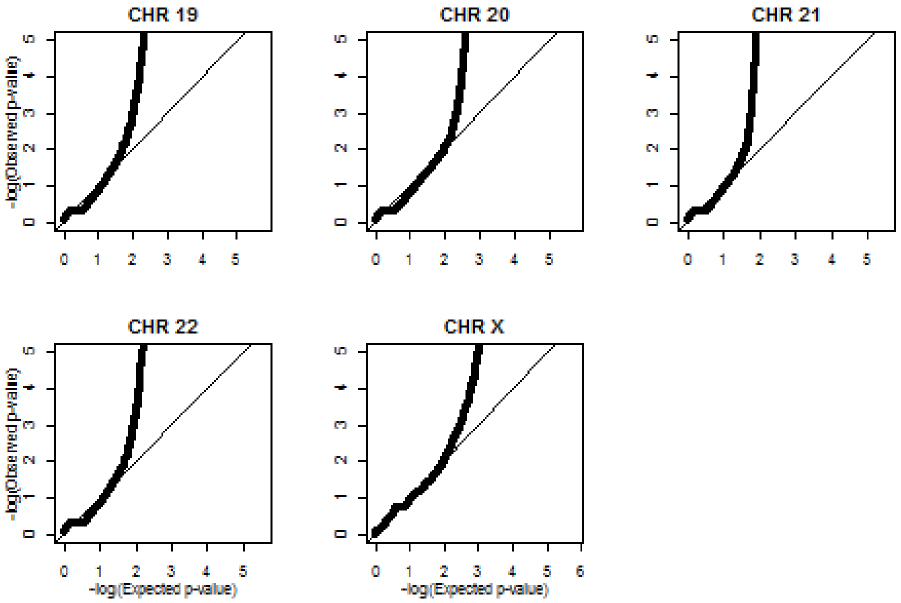

Figure S4: Supplementary Figure. QQ-plots of Exact HW p-values for chromosomes 19 through 23 against a uniform distribution.

Chr	#SNPs	%Mono	%HWE sig.	%HetExc	Median f	Median DP
1	6,042,635	85.27	0.29	73.54	-0.006	18073
2	6,681,362	85.78	0.24	62.95	-0.005	18004
3	5,583,595	85.26	0.31	50.31	-0.005	17989
4	5,426,389	85.08	0.25	58.30	-0.005	17558
5	5,006,108	85.69	0.20	70.32	-0.010	17977
6	4,790,920	84.28	0.85	21.78	-0.005	17872
7	4,204,599	85.29	0.30	54.06	-0.005	17810
8	4,374,143	85.89	0.22	68.39	-0.007	17991
9	3,294,178	85.24	0.23	68.64	-0.009	17993
10	3,669,704	85.04	0.30	65.61	-0.007	18102
11	3,716,843	85.58	0.30	55.93	-0.008	18132
12	3,547,602	85.15	0.28	61.90	-0.008	18011
13	2,691,685	85.02	0.24	52.78	-0.005	17551
14	2,516,367	85.13	0.34	51.73	-0.005	17972
15	2,193,923	85.31	0.22	83.45	-0.009	18273
16	2,411,903	85.89	0.30	82.44	-0.005	18205
17	2,063,337	85.65	0.40	81.23	-0.005	17865
18	2,146,584	85.12	0.24	61.38	-0.008	17898
19	1,552,728	84.75	0.42	55.68	-0.010	17127
20	1,707,286	85.73	0.24	77.20	-0.010	18320
21	997,277	84.96	0.29	54.67	-0.005	17721
22	950,560	84.98	0.37	57.38	-0.008	18041
Autosomes	75,569,728	85.31	0.31	55.67	-0.006	17941
X (all)	1,339,142	78.62	0.20			13487
X (fem)	1,339,142	80.80	0.12	71.29	-0.021	13487
Genome	76908870	85.19	0.31			17858

Table S1: Supplementary Table. Descriptive statistics for each chromosome and autosome-wide and genome-wide summaries of the JPT sample. Only variants with RS identifier having less than 5% missing values and *outside* simple repeat regions and segmental duplications are included. Table gives number of SNPs, percentage monomorphic markers, percentage significant markers in a HW Exact test with $\alpha = 0.001$ among the polymorphic markers, percentage of significant markers due to heterozygote excess, median of the inbreeding coefficient (f) for all polymorphic variants, median read depth (DP) for all polymorphic variants. Results for the X chromosome reported for an all-individuals test and for a females-only test.

B Appendix: Results for the YRI sample

B.1 Relationship missing values and HW for the YRI sample

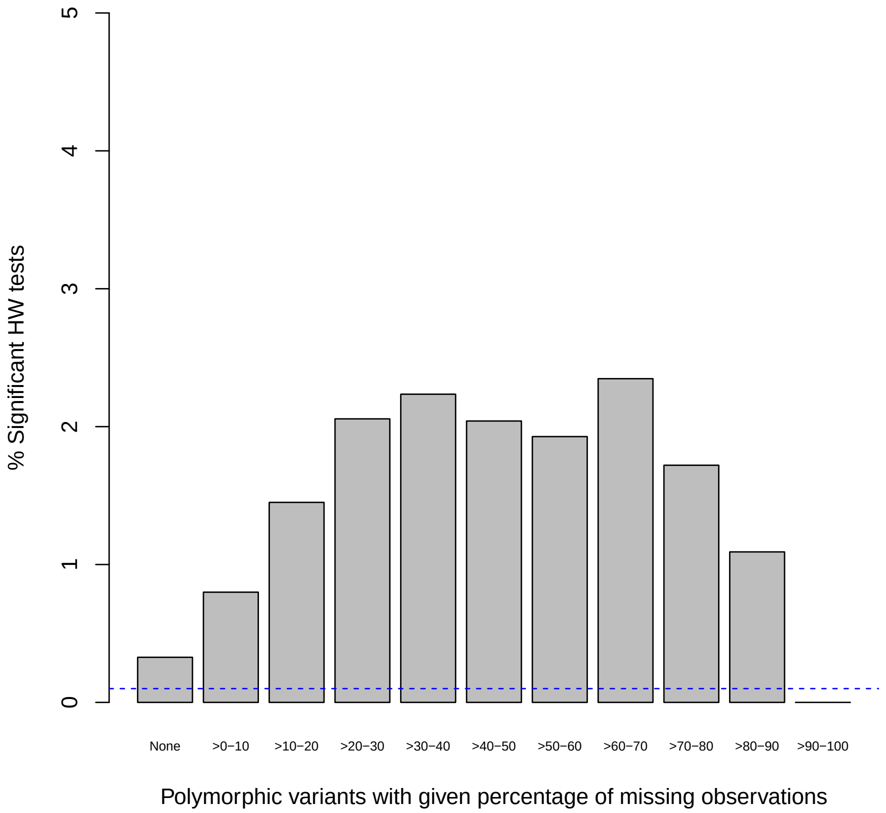

Figure S5: Percentage of significant HW tests for polymorphic autosomal variants of the YRI sample as a function of the percentage of missing values at $\alpha = 0.001$. The horizontal dashed line corresponds to the HapMap exclusion threshold.

B.2 Manhattan plot for the YRI sample

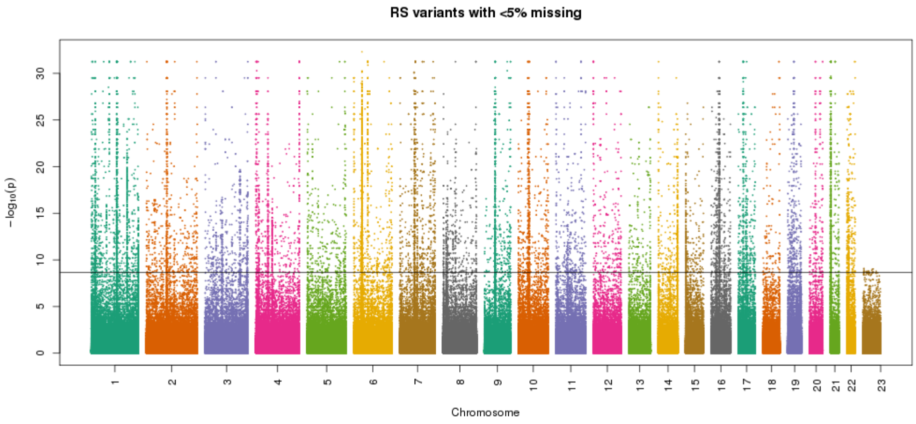

Figure S6: Manhattan plot of exact mid p-values for Hardy-Weinberg equilibrium of the YRI sample. The horizontal line corresponds to the Bonferroni significance threshold ($-log_{10}(0.05/22338807) = 8.7$, using only polymorphic autosomal variants).

B.3 Descriptive statistics for the YRI sample

Chr	#SNPs	%Mono	%HWE sig.	%HetExc	Median f	Median DP
1	6,445,632	72.76	0.42	56.67	-0.009	18037
2	7,057,087	73.06	0.25	56.30	-0.009	17974
3	5,811,885	72.51	0.26	39.68	-0.009	17957
4	5,711,988	71.55	0.32	41.76	-0.009	17548
5	5,247,197	72.38	0.19	59.13	-0.009	17949
6	5,005,316	71.64	0.56	25.47	-0.009	17863
7	4,699,755	71.94	0.31	50.33	-0.009	17768
8	4,581,191	72.65	0.26	48.95	-0.009	17968
9	3,548,192	72.63	0.37	50.22	-0.009	17928
10	3,977,918	72.07	0.31	57.70	-0.009	18074
11	4,031,235	72.61	0.26	52.35	-0.009	18114
12	3,761,426	72.54	0.21	59.30	-0.009	17980
13	2,847,601	71.80	0.18	54.55	-0.009	17526
14	2,645,783	72.66	0.47	45.70	-0.009	17945
15	2,416,028	72.61	0.37	58.76	-0.009	18243
16	2,688,407	73.37	0.43	69.20	-0.009	18210
17	2,320,340	72.95	0.36	67.63	-0.009	17806
18	2,259,220	72.10	0.17	68.56	-0.009	17863
19	1,824,822	71.58	0.56	42.19	-0.009	17144
20	1,806,662	72.58	0.20	75.97	-0.009	18302
21	1,101,242	71.22	0.78	78.72	-0.009	17737
22	1,099,340	72.19	0.58	40.33	-0.009	17961
Autosomes	80,888,267	72.38	0.33	50.88	-0.009	17913
X (all)	1,446,901	54.44	0.18			13444
X (fem)	1,446,901	57.52	0.11	75.76	-0.019	13444
Genome	82,335,168	72.07	0.32			17816

Table S2: Descriptive statistics for each chromosome and autosome-wide and genome-wide summaries of the YRI sample: number of SNPs (with RS identifier and with less than 5% missing values), percentage monomorphic markers, percentage significant markers in a HW Exact test with $\alpha = 0.001$ among the polymorphic markers, percentage of significant markers due to heterozygote excess, median of the inbreeding coefficient (f) for all polymorphic variants, median read depth (DP) for all polymorphic variants. Results for the X chromosome reported for an all-individuals test and for a females-only test.

B.4 MHC region of the YRI sample

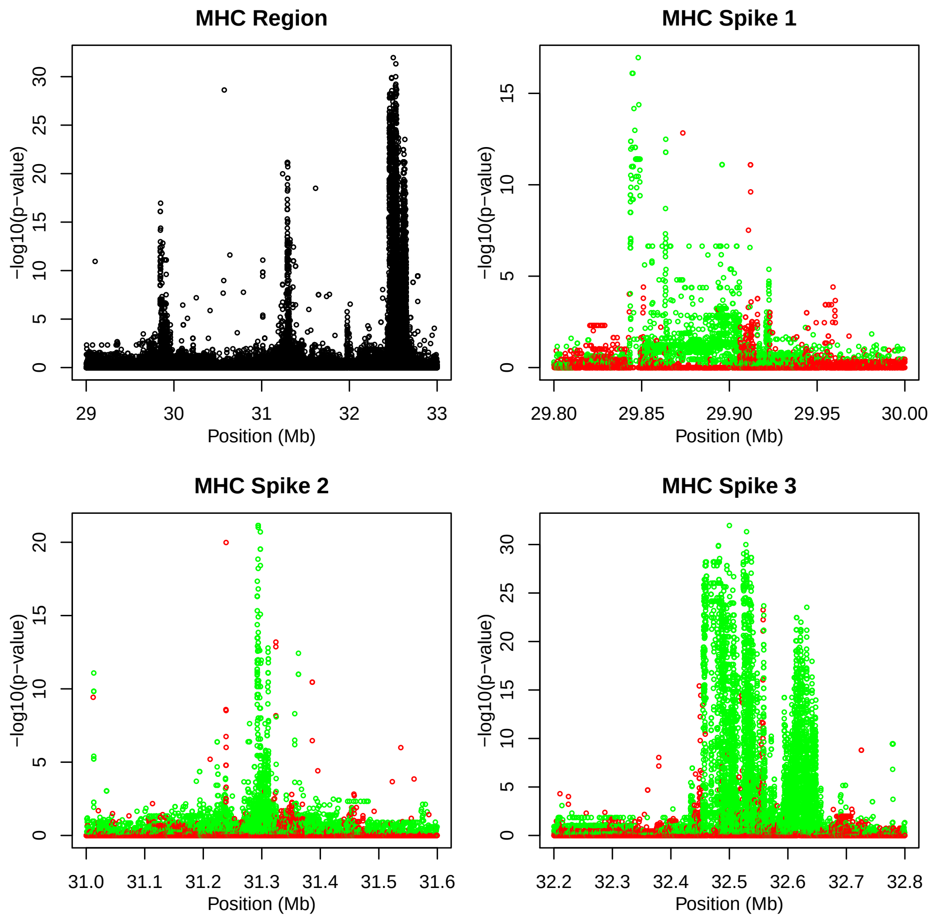

Figure S7: Supplementary Figure. A: Hardy-Weinberg track showing the exact mid p-values of tests for disequilibrium for each variant in the MHC region on chromosome 6 for the Yoruba sample ($n = 107$). B,C,D: Plots of the exact p-values for the observed spikes colored according to the sign of the inbreeding coefficient (green $f > 0$, red $f < 0$).

B.5 Descriptive statistics for areas of the YRI sample

	#SNPs	%Mono	%Sig	%HetExc	Med(f)	Med(DP)
CHR 6	5,005,316	71.64	0.56	25.47	-0.009	17863
MHC	136,099	53.24	7.93	3.51	-0.005	17179
outside MHC	4,869,217	72.16	0.21	64.13	-0.009	17886
HLA-A	342	25.44	3.14	75.00	-0.060	16231
HLA-B	302	33.11	3.47	42.86	-0.005	12445
HLA-C	327	23.55	4.40	72.73	0.029	14454
HLA-DPA1	943	30.97	0.15	0.00	-0.005	18650
HLA-DPB1	758	36.15	5.79	0.00	-0.005	17948
HLA-DQA1	864	8.80	30.96	0.00	0.189	11896
HLA-DQB1	905	18.34	37.48	0.00	0.256	10826
HLA-DRB1	838	9.79	16.53	27.20	-0.009	11238
HLA-DRA	162	46.30	0.00		-0.003	19598
CHR 21	1,102,563	71.15	0.792	79.20	-0.009	17737
CHR 21 p.arm	23,453	78.43	21.787	77.50	-0.009	38631
CHR 21 q.arm	1,072,796	70.96	0.255	65.24	-0.009	17684
CHR 1 (Cen)	29,864	0.000	5.304	80.81	-0.009	19490
CHR 1 outside	6,421,563	73.038	0.345	51.14	-0.009	18019
CHR 2 (Cen)	13,572	0.000	3.190	89.15	-0.005	18762
CHR 2 outside	7,049,926	73.136	0.230	53.74	-0.009	17969
CHR 3 (Cen)	31,358	0.000	0.128	82.50	-0.009	17243
CHR 3 outside	5,786,048	72.841	0.268	40.16	-0.009	17971
CHR 4 (Cen)	16,777	0.000	2.748	89.59	-0.009	18948
CHR 4 outside	5,701,096	71.688	0.297	37.84	-0.009	17535
CHR 4 (4pTel)	4,730	74.86	3.95	100.00	-0.013	18561
CHR 10 (10pTel)	3,704	75.89	3.58	9.38	-0.005	17897
CHR 12 (12pTel)	2,162	73.03	12.52	97.26	-0.019	26593
CHR 14 (14pTel)	11,094	83.62	2.04	97.30	-0.005	23090

Table S3: Descriptive statistics for certain genomic areas of the YRI sample: number of SNPs, percentage monomorphic markers, percentage significant markers in a HW Exact test with $\alpha = 0.001$, percentage of significant markers due to heterozygote excess and medians of the inbreeding coefficient (f) and the read depth (DP).

B.6 P arm chromosome 21 YRI

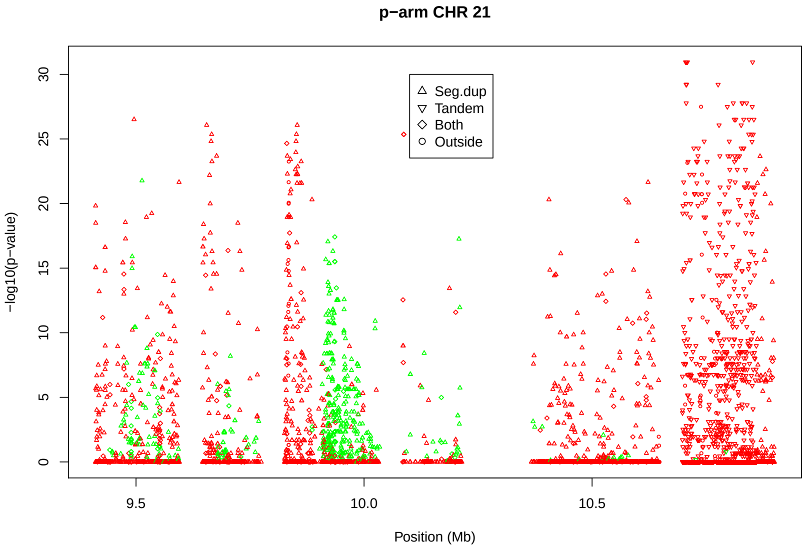

Figure S8: Plots of exact p-values on the p-arm of chromosome 21 of the YRI sample (green $f > 0$, red $f < 0$)

B.7 Centromeres of the YRI sample

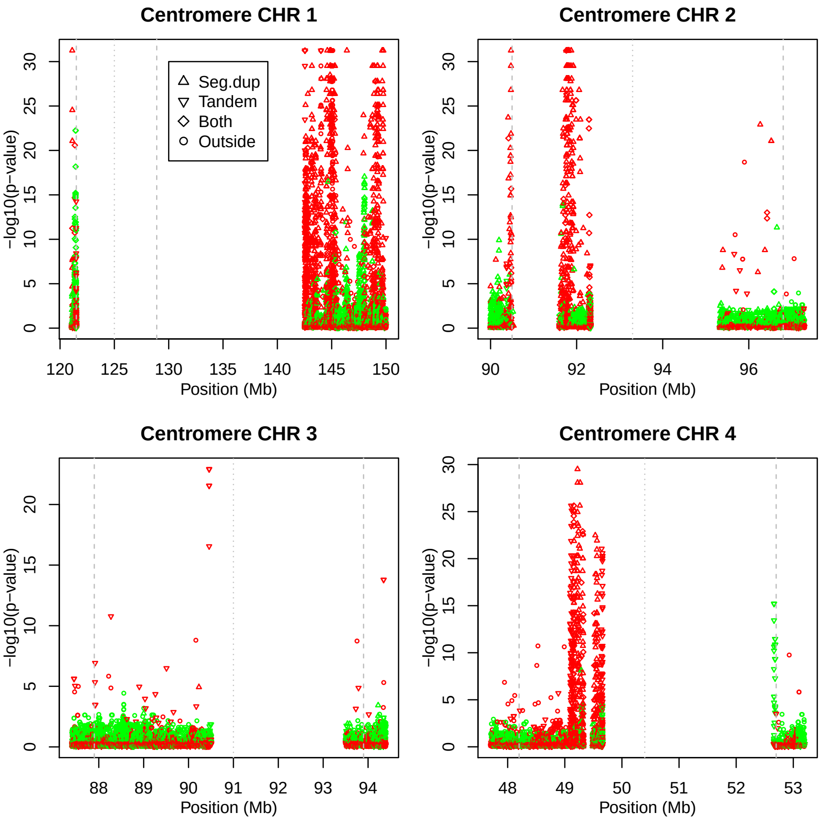

Figure S9: Plots of exact p-values around the centromeres of chromosomes 1 through 4 for the YRI sample. (green $f > 0$, red $f < 0$).

B.8 Telomeres of the YRI sample

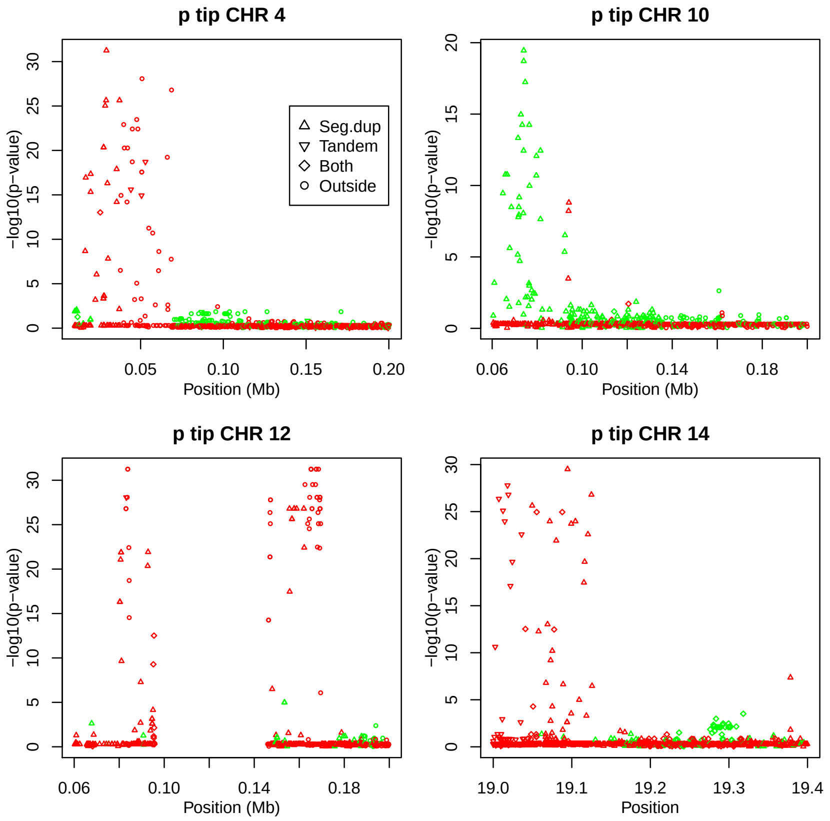

Figure S10: Plots of exact p-values at the start of chromosomes 4, 10, 12 and 14 of the YRI sample. (green $f > 0$, red $f < 0$).

B.9 X-chromosome of the YRI sample

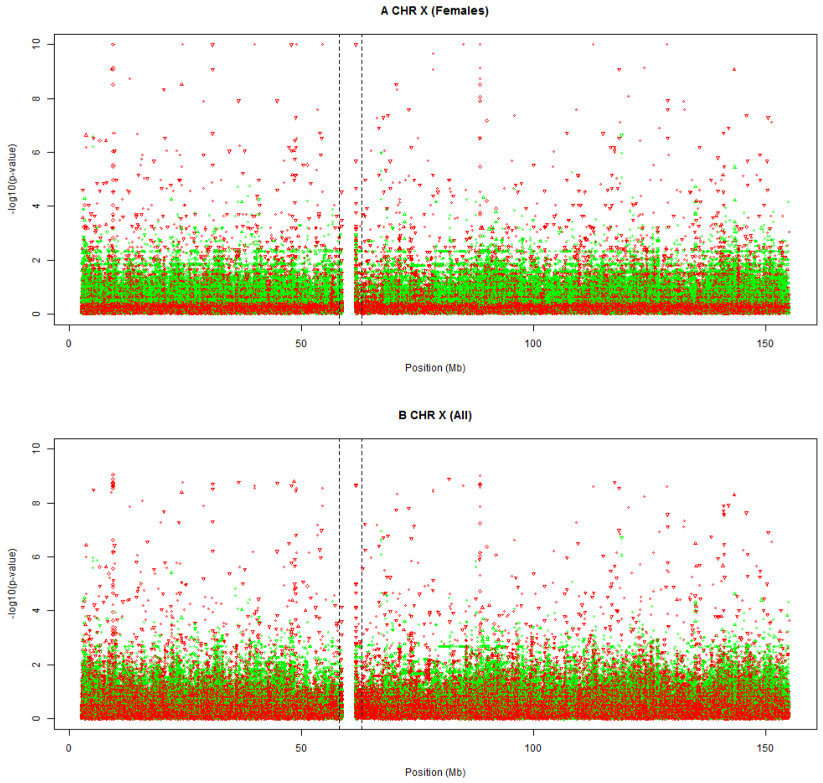

Figure S11: Plots of exact mid p-values of chromosome X for the YRI sample. A: Testing females only. B: testing males and females (green $f > 0$, red $f < 0$). Dashed vertical lines indicate the limits of the centromere region.

B.10 Horizontal bands of p-values for the YRI sample

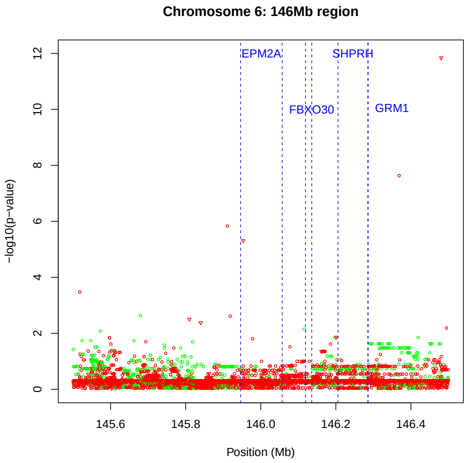

Figure S12: Plots of exact p-values at 145.5Mb-146.5Mb on chromosome 6 of the YRI sample (green $f > 0$, red $f < 0$) Monomorphic markers not shown.

.

B.11 Incidental spikes for the YRI sample

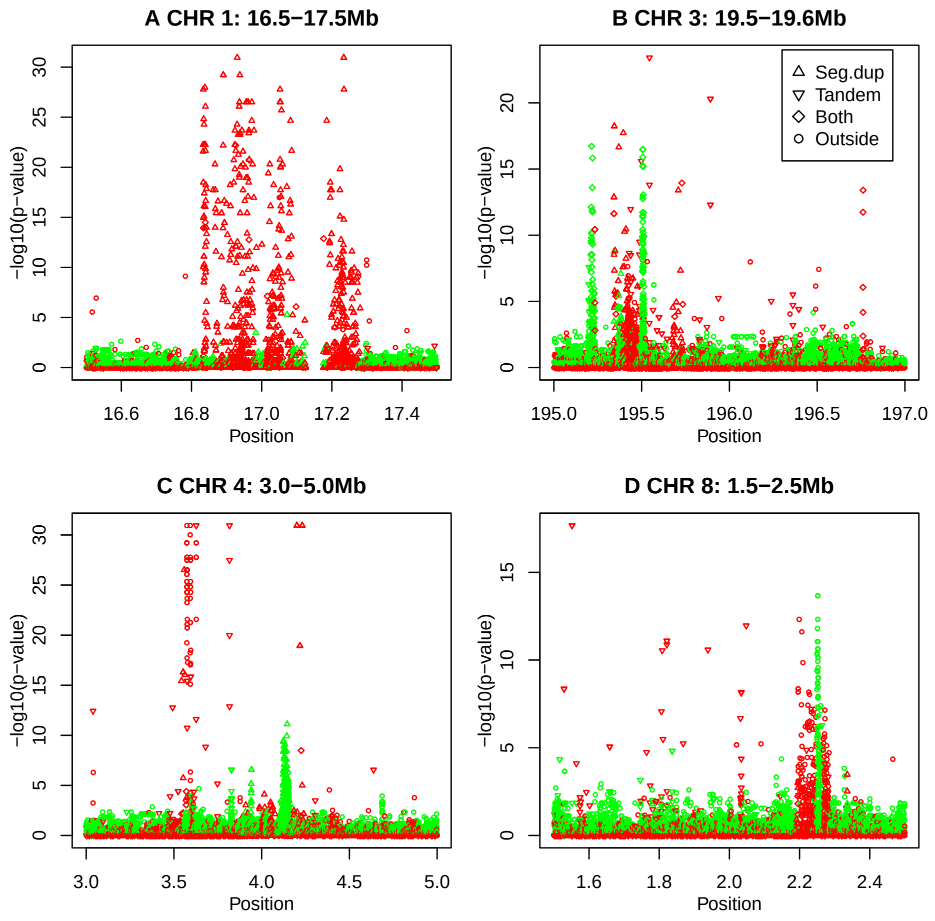

Figure S13: A: Hardy-Weinberg track showing the exact mid p-values of tests for disequilibrium for each variant in the MHC region on chromosome 6. B,C,D: Plots of the exact p-values for the observed spikes colored according to the sign of the inbreeding coefficient (green $f > 0$, red $f < 0$), annotated with HLA class I and II genes. Plotting symbols indicate if a variant is inside a segmental duplication, inside a tandem repeat, inside both, or outside such regions.

B.12 Relation with read depth for the YRI sample

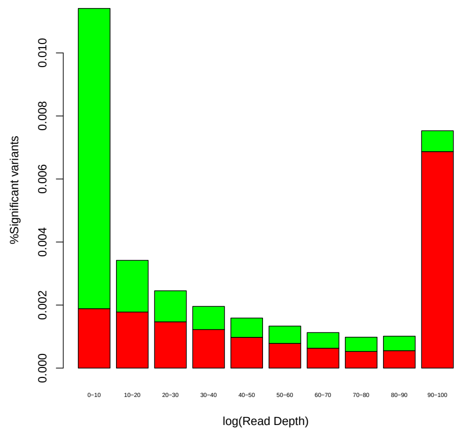

Figure S14: Percentage of significant HWD as a function of read depth for the YRI sample (green $f > 0$, red $f < 0$).

B.13 Relation with segmental duplications and with simple repeat regions for the YRI sample

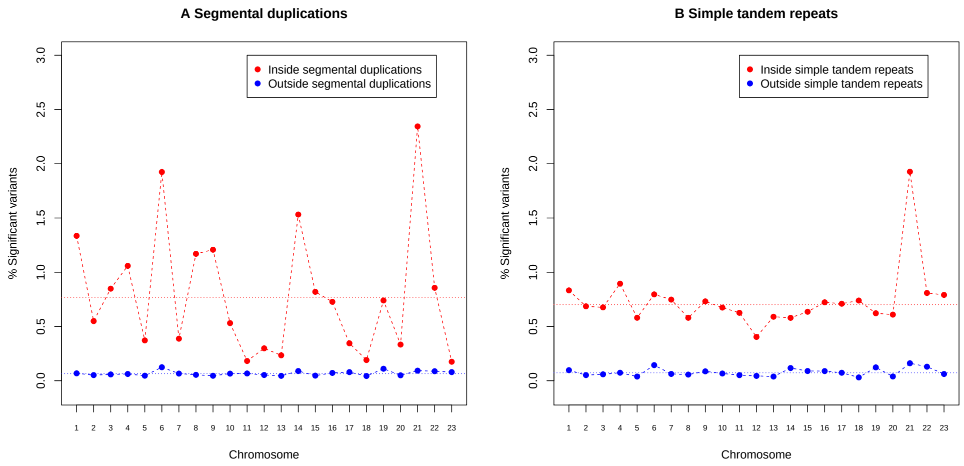

Figure S15: A: Percentage of significant HWD in and outside segmental duplications for each chromosome. B: Percentage of significant HWD in and outside simple tandem repeats for each chromosome. Dotted horizontal lines represent the overall autosomal rate.

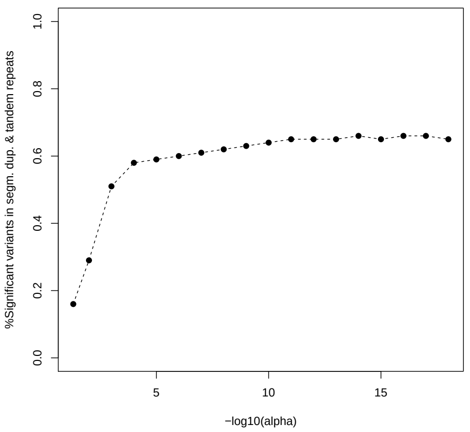

Figure S16: Percentage of significant HWD in and outside segmental duplications for each chromosome as a function of the significance threshold (α)

B.14 Appendix: Supplementary material for the YRI sample

B.14.1 Exact p-value as a function of MAF

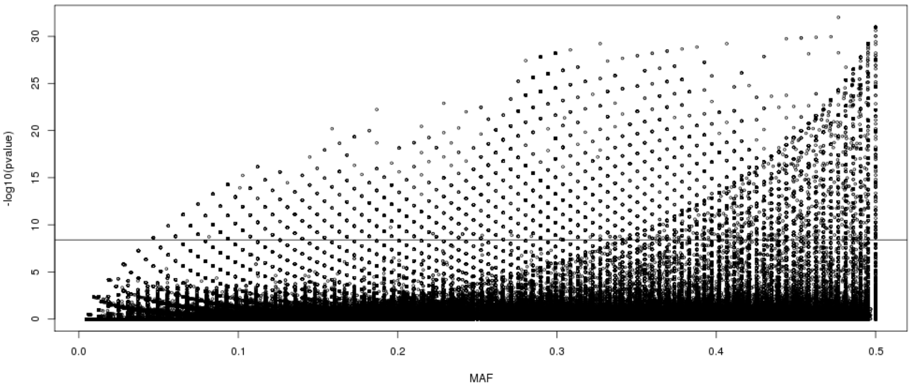

Figure S17: Supplementary Figure. Exact HW p-value as a function of the minor allele frequency (MAF) for 12.4 million polymorphic variants of the YRI population.

Chr	#SNPs	%Mono	%HWE sig.	%HetExc	Median f	Median DP
1	6,040,016	73.08	0.19	51.46	-0.009	18068
2	6,678,323	73.39	0.14	46.40	-0.009	18014
3	5,581,156	72.83	0.17	35.58	-0.009	17992
4	5,423,685	71.96	0.16	40.26	-0.009	17577
5	5,003,610	72.72	0.12	48.61	-0.009	17992
6	4,788,576	72.11	0.38	24.07	-0.009	17907
7	4,202,648	72.43	0.15	47.94	-0.009	17809
8	4,372,054	72.93	0.14	45.88	-0.009	18005
9	3,292,691	72.90	0.12	56.70	-0.009	17990
10	3,668,026	72.41	0.16	59.02	-0.009	18116
11	3,715,147	72.96	0.17	45.76	-0.009	18156
12	3,546,021	72.93	0.14	52.34	-0.009	18020
13	2,690,319	72.23	0.12	43.86	-0.009	17565
14	2,515,198	72.93	0.28	47.26	-0.009	17988
15	2,192,920	73.00	0.14	59.70	-0.009	18288
16	2,410,718	73.61	0.17	55.75	-0.009	18226
17	2,062,343	73.43	0.20	65.72	-0.009	17884
18	2,145,582	72.50	0.10	59.93	-0.009	17907
19	1,551,865	72.29	0.26	45.42	-0.009	17159
20	1,706,515	72.96	0.12	63.72	-0.009	18343
21	996,674	71.38	0.12	71.52	-0.009	17726
22	950,051	73.02	0.21	40.33	-0.009	18044
Autosomes	75534138	72.76	0.17	45.35	-0.009	17954
X (all)	1338295	55.21	0.13			13488
X (females)	1338295	58.32	0.06	62.78	-0.019	13488
Genome	76872433	72.45	0.17			17860

Table S4: Supplementary Table. Descriptive statistics for each chromosome and autosome-wide and genome-wide summaries of the YRI sample. Only variants with RS identifier having less than 5% missing values and *outside* simple repeat regions and segmental duplications are included. Table gives number of SNPs, percentage monomorphic markers, percentage significant markers in a HW Exact test with $\alpha = 0.001$ among the polymorphic markers, percentage of significant markers due to heterozygote excess, median of the inbreeding coefficient (f) for all polymorphic variants, median read depth (DP) for all polymorphic variants. Results for the X chromosome reported for an all-individuals test and for a females-only test.

B.14.2 QQ-plots of Exact p-values for each chromosome for the YRI sample

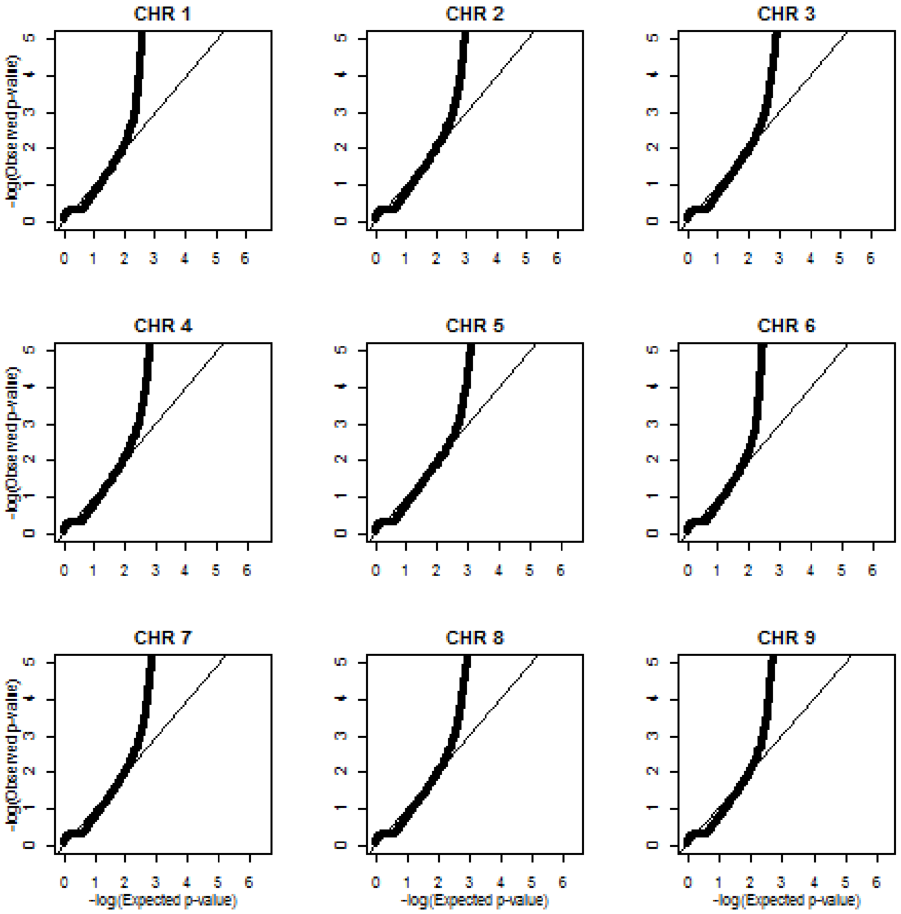

Figure S18: Supplementary Figure. QQ-plots of Exact HW p-values for chromosomes 1 through 9 against a uniform distribution.

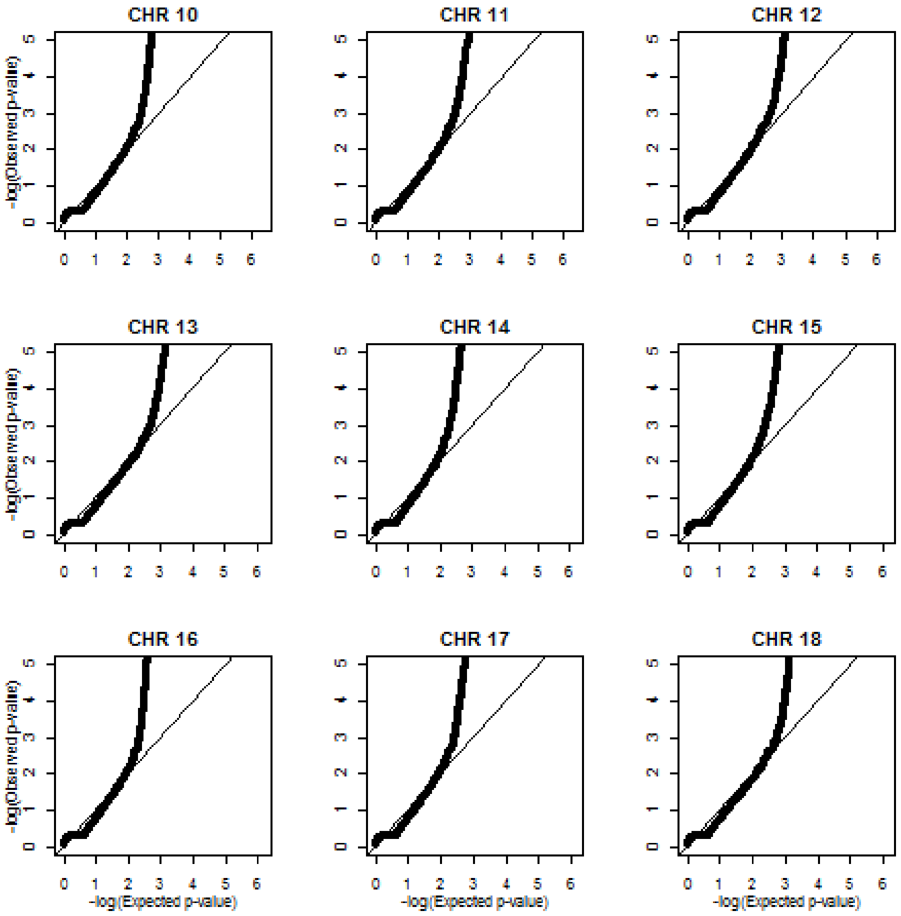

Figure S19: Supplementary Figure. QQ-plots of Exact HW p-values for chromosomes 10 through 18 against a uniform distribution.

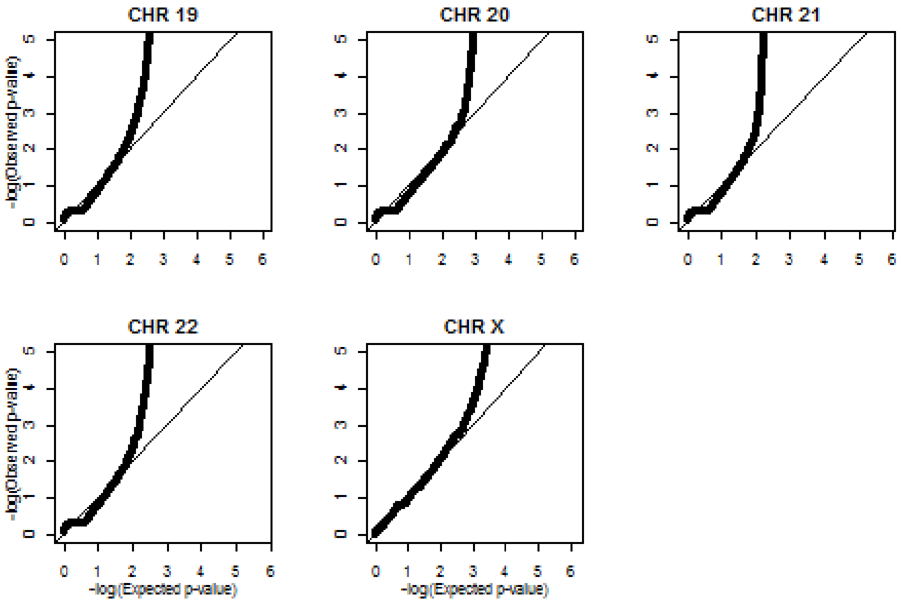

Figure S20: Supplementary Figure. QQ-plots of Exact HW p-values for chromosomes 19 through 23 against a uniform distribution.
